# Column Generation for Action-Based Group Handover Optimization in Large-Scale LEO Satellite Networks

**DOI:** 10.3390/s26196309

**Published:** 2026-10-06

**Authors:** Yang Liu, Wen Liu, Wenliang Lin, Heng Kang, Zhongliang Deng

**Affiliations:** School of Electronic Engineering, Beijing University of Posts and Telecommunications, Beijing 100876, China; liuyang13@bupt.edu.cn (Y.L.); charterlin@bupt.edu.cn (W.L.); kangheng@bupt.edu.cn (H.K.); a20251121@bupt.edu.cn (Z.D.)

**Keywords:** LEO satellite networks, group handover, executable actions, column generation, heuristic pricing

## Abstract

Low Earth orbit (LEO) satellite constellations are an important component of non-terrestrial networks (NTNs) in future sixth-generation (6G) systems, owing to their wide coverage and low propagation delay. However, due to the high-speed orbital movement of LEO satellites, the serving satellites of massive ground terminals change frequently, resulting in frequent and highly concurrent handover requests. Existing group handover methods usually follow a two-stage paradigm that first forms terminal groups according to similarity features and then performs target satellite selection or handover scheduling for the pre-formed groups. Such a grouping-first strategy may restrict the group handover action space and exclude potentially better terminal-satellite-slot combinations from consideration. To address this issue, this paper proposes an executable-action-based group handover paradigm, where terminal grouping, target satellite selection, and handover-slot assignment are unified in the action construction process. Based on this formulation, the group handover problem is modeled as a large-scale combinatorial optimization problem. To solve it efficiently, this paper proposes a Group Handover Action Column Generation (GHACG) algorithm. In the column generation framework, the algorithm starts from a restricted set of executable actions and iteratively solves the corresponding restricted master problem. The obtained dual prices are then used to guide heuristic pricing to generate high-value executable action columns, and an integer action recovery procedure is designed to obtain a feasible group handover scheme. Simulation results show that GHACG outperforms representative single-user and group handover baselines in terms of handover success rate, signaling overhead, number of group handover actions, handover rate, and handover objective value J. Under the largest simulated terminal scale of $1.2\times 10^{5}$, GHACG improves the handover success rate by 11.17% compared with the best-performing baseline.

## 1. Introduction

Non-terrestrial networks (NTNs) have become a key component of future sixth-generation (6G) communication systems as an important complement to terrestrial cellular communications. Low Earth orbit (LEO) satellite communication networks, owing to their relatively low propagation delay, flexible deployment, and wide-area coverage, serve as an important infrastructure for supporting global ubiquitous access, emergency communications, natural disaster monitoring, sensor data transmission, remote-area coverage, and space-air-ground integrated networks [1,2,3,4,5,6].

LEO satellites generally operate in near-Earth orbits with altitudes of approximately 500–2000 km. Due to their high orbital velocity, the visibility duration between a LEO satellite and a ground terminal is usually limited, and the serving satellite of a terminal may change frequently over time. As a result, ground terminals need to perform frequent inter-satellite handovers to maintain service continuity. For example, in the Iridium satellite communication system, a ground terminal may experience a handover approximately every 10 min on average. Frequent handovers not only increase the risk of call drops and service interruption, but also introduce additional signaling overhead, access contention, and resource blocking [7,8].

Compared with terrestrial cellular base stations, a single LEO satellite usually covers a much larger geographical area. Therefore, coverage variations caused by satellite mobility may trigger a large number of ground terminals to initiate handovers within a short time interval. According to the prediction in 3GPP TR 38.821, when the user density reaches 33.36 users/km^2^, the average handover rate can be as high as 9912 users/s [9]. This indicates that handover management in LEO satellite networks is not merely a single-terminal link maintenance problem, but a network-level multi-terminal coordination problem.

Existing studies have explored several approaches to single-terminal handover optimization in LEO satellite networks. Rule-based and geometry-based methods determine handover triggering and target satellite selection using elevation angle, terminal-satellite distance, link quality, remaining visibility time, and threshold or hysteresis conditions [10,11,12,13]. These methods offer simple models, clear physical interpretations, and practical applicability, but they rely primarily on instantaneous criteria and thus may not fully exploit the predictability of satellite motion for handover optimization over a longer time horizon. Multi-attribute decision-making methods evaluate candidate satellites using link quality, satellite load, delay, remaining visibility time, service requirements, and user preferences [14,15,16]. These methods allow more flexible handover decisions by considering multiple factors, but their performance can be sensitive to weight selection and utility-function design. Some studies use graph matching, network flow, integer programming, or Markov decision processes to model and optimize user-satellite association and handover decisions [17,18,19,20]. These methods explicitly model resource constraints and the coupling among handover decisions, but their computational cost can grow substantially with problem size, making it difficult to efficiently handle large-scale concurrent handovers.

Recent studies have also introduced reinforcement learning into LEO satellite handover decision-making. These methods usually take connection status, link quality of candidate satellites, remaining visibility time, and network load as state inputs, define connection maintenance or target satellite selection as actions, and learn long-term handover policies using DQN, DDQN, Actor-Critic, PPO, or multi-agent reinforcement learning algorithms [21,22,23,24,25,26,27,28,29,30,31]. Such methods have the potential to learn long-term decision policies in dynamic environments. However, their performance usually depends on a large number of training samples, careful parameter tuning, and the consistency between training and deployment scenarios, which makes policy interpretability, convergence stability, and cross-scenario generalization remain major challenges.

Some studies further consider the joint optimization of handover decisions with beam management, power allocation, spectrum sharing, information freshness, virtual network function mapping, and QoS provisioning, aiming to improve the overall resource utilization and service performance of LEO satellite networks [32,33,34,35,36,37,38,39,40]. By integrating handover control with other resource management and service optimization tasks, these methods provide a more comprehensive network-level optimization perspective. However, such joint optimization usually involves multiple types of decision variables, heterogeneous objectives, and coupled constraints, which significantly increases the modeling and solution complexity. In addition, their performance often depends on the adopted scenario assumptions, weighting coefficients, and parameter settings.

Overall, the above single-terminal handover studies have improved LEO satellite handover performance from the perspectives of handover triggering, target satellite selection, long-term decision-making, and joint resource optimization. Nevertheless, they generally take a single terminal or mutually independent handover requests as the basic decision object. As a result, they cannot effectively address large-scale concurrent handover scenarios.

With the large-scale deployment of LEO satellite constellations and the growing satellite access demand for Internet services and distributed sensing applications, handover management in LEO satellite networks is evolving from individual handovers in sparse-user scenarios to large-scale coordinated handovers in dense-user scenarios. Accordingly, group handover has attracted increasing research attention in recent years. Existing studies on LEO satellite group handover usually start from user grouping. A typical class of methods forms user groups according to similarity features such as user location, beam coverage relationship, serving satellite, candidate target satellite, or service attributes, and then checks the feasibility of the formed groups based on constraints such as common target satellite availability, remaining service time, satellite capacity, or bandwidth resources [41,42,43]. These methods reduce signaling overhead and scheduling complexity by aggregating users with similar handover characteristics. However, user groups are fixed before target satellite selection and resource allocation, preventing subsequent scheduling from considering alternative groupings that may better match the available satellite resources.

Another category of studies introduces prediction into group handover scheduling. These methods exploit historical handover sequences, terminal locations, mobility patterns, measurement reports, or the predictability of satellite movement to predict the target satellite, handover time, or future service state of user groups [44,45]. Based on such prediction information, group handover scheduling and multi-layer handover process design can be further performed to reduce concurrent handover congestion and improve system performance. However, their performance is closely related to the accuracy of handover prediction and network-state estimation.

Some studies address group handover scheduling from the perspective of graph-based optimization or mathematical programming. They model the relationships among user groups, candidate satellites, and handover slots as bipartite graphs, multi-layer graphs, auxiliary graphs, network flow models, or integer programming problems, so as to perform target satellite assignment, handover path planning, load balancing, and scheduling optimization [46,47,48]. These methods effectively optimize the matching and scheduling of predefined user groups and satellite resources. However, because group formation is not jointly optimized with scheduling, the resulting solution remains constrained by the initial grouping. In addition, some studies jointly optimize user association and handover decisions by considering signaling overhead, handover frequency, routing, or satellite load conditions [49,50]. These methods usually adopt heuristic algorithms such as simulated annealing for solution search. Although they can incorporate multiple performance metrics, heuristic search methods may incur high computational overhead, and their performance can be sensitive to the cooling schedule and parameter configuration, leading to slow convergence or unstable solutions in large-scale scenarios. Some studies also formulate user-group or multi-user handover processes as MDP, DEC-MDP, or reinforcement learning problems to learn dynamic handover, target satellite selection, and beam selection strategies [51,52]. These methods have the capability to learn adaptive policies under time-varying LEO satellite network conditions. However, they usually require a large number of training samples and still face challenges in policy interpretability and convergence stability. Table 1 summarizes the technical approaches, decision entities, and main methods of representative handover studies.

In summary, most existing LEO satellite group handover methods follow a two-stage paradigm of “**grouping first and scheduling later**”. Once the user groups are fixed, the subsequent optimization can only search over the action set induced by these groups. As a result, some potentially better group handover actions formed by different combinations of users, target satellites, and handover slots may never be considered, leading to group handover action-space loss.

To address this issue, this paper formulates large-scale group handover in LEO satellite networks as an optimization problem over executable group handover actions. The main contributions are summarized as follows.

(a) First, to the best of our knowledge, this paper is the first to formulate LEO satellite group handover as an executable action optimization problem. By selecting executable actions, the model jointly determines terminal groups and their satellite-slot assignments, allowing potentially better executable handover actions excluded by fixed pre-grouping to be considered in the optimization.
(b) Second, in the executable-action model, the number of candidate action variables grows exponentially with the number of handover terminals, and the resulting large-scale action selection problem is NP-hard. To solve this problem, this paper proposes a Group Handover Action Column Generation (GHACG) algorithm. GHACG uses the dual prices obtained from the linear relaxation of the restricted master problem to guide action generation, turning undirected action exploration into reduced-cost-guided search. Since the column generation process may produce fractional solutions, an integer action recovery procedure is designed to transform the generated action columns into executable group handover schedules. The algorithm enables efficient group handover optimization in large-scale LEO satellite networks with massive ground terminals.
(c) To validate the effectiveness of the proposed GHACG algorithm, system-level simulations based on realistic satellite ephemeris data are conducted. Representative single-user and group handover baselines, including several group handover schemes reproduced from published studies, are used for comparison. The simulation results show that GHACG outperforms these baselines in terms of handover success rate, signaling overhead, number of group handover actions, handover rate, and handover objective value. Under the largest simulated terminal scale, GHACG improves the handover success rate by 11.17% compared with the best-performing baseline.

The remainder of this paper is organized as follows. Section 2 presents the system model and problem formulation and provides a theoretical analysis of the action-space and performance losses induced by fixed pre-grouping. Section 3 describes the proposed GHACG algorithm. Section 4 presents the simulation results and analysis. Finally, Section 5 concludes the paper.

## 2. System Model and Problem Formulation

### 2.1. Network Scenario and Link Model

This paper considers a large-scale LEO satellite access network serving massive ground terminals, which are denoted as terminals for simplicity. Compared with the high orbital mobility of LEO satellites, the mobility of ground terminals is neglected in this study.

Let $S=\left\{1,2,\ldots ,M\right\}$ denote the set of LEO access satellites, where s∈S represents one satellite. Let $U^{ho}=\left\{1,2,\ldots ,N\right\}$ denote the set of terminals that require handover within the current decision window, where $i\in U^{ho}$ represents one handover terminal. The system time is divided into T equal-length slots with slot duration Δt, and the slot set is denoted by $T=\left\{1,2,\ldots ,T\right\}$, where τ∈T represents a handover decision slot. Since LEO satellite motion is much faster than ground terminal mobility, the positions of ground terminals are assumed to remain unchanged within one decision window.

For terminal i and satellite s, let $d_{i,s}\left(\tau \right)$ and $e_{i,s}\left(\tau \right)$ denote the geometric distance and the access elevation angle in slot τ, respectively. The access elevation angle $e_{i,s}\left(\tau \right)$ is defined as the angle between the line-of-sight direction from terminal i to satellite s and the local horizontal plane at the terminal location. A satellite link budget is adopted to evaluate link quality by accounting for the transmission, propagation, and reception characteristics of the satellite link [53]. Following the 3GPP NTN channel model [7], the total propagation loss between satellite s and terminal i in slot τ is decomposed as

(1) $$L_{i,s}^{tot}\left(\tau \right)=L_{i,s}^{b}\left(\tau \right)+L_{i,s}^{gas}\left(\tau \right)+L_{i,s}^{scin}\left(\tau \right)+L_{i,s}^{BE}\left(\tau \right)+L_{i,s}^{rain}\left(\tau \right)+L_{i,s}^{cloud}\left(\tau \right).$$

where $L_{i,s}^{b}\left(\tau \right)$ denotes the basic path loss, which is given by

(2) $$L_{i,s}^{b}\left(\tau \right)=L_{i,s}^{FS}\left(\tau \right)+L_{i,s}^{SF}\left(\tau \right)+L_{i,s}^{CL}\left(\tau \right).$$

where $L_{i,s}^{FS}\left(\tau \right)$, $L_{i,s}^{SF}\left(\tau \right)$, and $L_{i,s}^{CL}\left(\tau \right)$ denote the free-space path loss, shadow fading loss, and clutter loss, respectively; and $L_{i,s}^{gas}\left(\tau \right)$, $L_{i,s}^{scin}\left(\tau \right)$, $L_{i,s}^{BE}\left(\tau \right)$, $L_{i,s}^{rain}\left(\tau \right)$, and $L_{i,s}^{cloud}\left(\tau \right)$ denote atmospheric gas attenuation, tropospheric scintillation loss, building-entry loss, rain attenuation, and cloud attenuation, respectively. The shadow fading term is modeled in the dB domain as $L_{i,s}^{SF}\left(\tau \right)∼N\left(0,\sigma_{SF}^{2}\right)$. The main simulations consider outdoor terminals under LOS and clear-sky conditions. Therefore, $L_{i,s}^{CL}\left(\tau \right)=L_{i,s}^{BE}\left(\tau \right)=L_{i,s}^{rain}\left(\tau \right)=L_{i,s}^{cloud}\left(\tau \right)=0.$ The free-space path loss, shadow fading, atmospheric gas attenuation, and tropospheric scintillation loss are retained in the main simulations. In the additional robustness simulations, clutter loss is included under NLOS conditions, while rain and cloud attenuation are included under adverse-weather conditions.

The free-space path loss is determined by the propagation distance and carrier frequency, and is given by

(3) $$L_{i,s}^{FS}\left(\tau \right)=20\log_{10}\left(\frac{4\pi f_{c}d_{i,s}\left(\tau \right)}{c}\right),$$

where $f_{c}$ is the carrier frequency and c is the speed of light. The atmospheric gas attenuation is calculated as

(4) $$L_{i,s}^{gas}\left(\tau \right)=\frac{A_{zenith}\left(f_{c}\right)}{{\sin e}_{i,s}\left(\tau \right)}$$

where $A_{zenith}\left(f_{c}\right)$ is the zenith atmospheric attenuation at carrier frequency $f_{c}$. Since the carrier frequency is 20 GHz, $L_{i,s}^{scin}\left(\tau \right)$ represents tropospheric scintillation loss and is determined from the elevation-dependent reference values of the 3GPP NTN channel model.

Let ${EIRP}_{s}$ denote the effective isotropic radiated power of satellite s, and let $G_{i}$ denote the receive antenna gain of terminal i. The received signal power at terminal i from satellite s is then given by

(5) $$P_{i,s}^{rx}\left(\tau \right)={EIRP}_{s}+G_{i}-L_{i,s}^{tot}\left(\tau \right).$$

In a multi-satellite coverage scenario, a terminal may be covered by multiple satellites simultaneously. Considering the aggregate interference caused by co-channel satellite links, the signal-to-interference-plus-noise ratio (SINR) of terminal i when accessing satellite s in slot τ is expressed as

(6) $$\gamma_{i,s}\left(\tau \right)=\frac{p_{i,s}^{rx}\left(\tau \right)}{I_{i,s}\left(\tau \right)+N_{0}B\;10^{F_{i}/10}}.$$

where $p_{i,s}^{rx}\left(\tau \right)=10^{P_{i,s}^{rx}\left(\tau \right)/10}$ is the received signal power in W, $I_{i,s}\left(\tau \right)$ is the aggregate interference power in W, $N_{0}$ is the thermal noise power spectral density in W/Hz, B is the channel bandwidth in Hz, and $F_{i}$ is the receiver noise figure of terminal i in dB.

Based on the Shannon rate relationship, the achievable spectral efficiency of terminal i when accessing satellite s in slot τ is given by

(7) $$\eta_{i,s}\left(\tau \right)=\log_{2}\left(1+\gamma_{i,s}\left(\tau \right)\right).$$

Let $R_{i}^{req}$ denote the service rate requirement of terminal i. The equivalent bandwidth required to satisfy this rate requirement when terminal i accesses satellite s in slot τ can be expressed as

(8) $$b_{i,s}\left(\tau \right)=\frac{R_{i}^{req}}{\eta_{i,s}\left(\tau \right)}.$$

Let $C_{s}\left(\tau \right)$ denote the residual access bandwidth of satellite s in slot τ. It should be noted that $C_{s}\left(\tau \right)$ does not represent the total system bandwidth of satellite s. Instead, it denotes the residual access bandwidth available for admission in the current decision slot.

According to the elevation visibility, link availability and resource admissibility, the candidate target satellite set of terminal i in slot τ is defined as

(9) $$S_{i}\left(\tau \right)=\left\{s\in S|e_{i,s}\left(\tau \right)\geq e_{min},\gamma_{i,s}\left(\tau \right)\geq \gamma_{min},b_{i,s}\left(\tau \right)\leq C_{s}\left(\tau \right)\right\},$$

where $e_{min}$ is the minimum access elevation angle, and $\gamma_{min}$ is the link availability threshold. This definition indicates that a candidate target satellite must be visible from the terminal, provide an available access link, and have sufficient residual bandwidth to admit the terminal. The construction and feasibility evaluation of group handover actions are performed based on this candidate satellite set.

### 2.2. Executable Group Handover Action Model

Unlike conventional two-stage group handover methods that first form terminal groups according to similarity and then determine the target satellite for each fixed group, this paper takes an executable group handover action as the basic optimization unit. In such methods, the predefined grouping result may restrict the subsequent target-satellite selection and cause a loss of feasible action space. To avoid this limitation, terminal grouping, target satellite selection, and handover-slot determination are jointly represented at the action level. A group handover action is defined as

(10) $$a=\left(G_{a},s_{a},\tau_{a}\right),$$

where $G_{a}\subseteq U^{ho}$ is the set of terminals involved in action a, $s_{a}\in S$ is the target satellite, and $\tau_{a}\in T$ is the handover execution slot.

**Figure 1:**
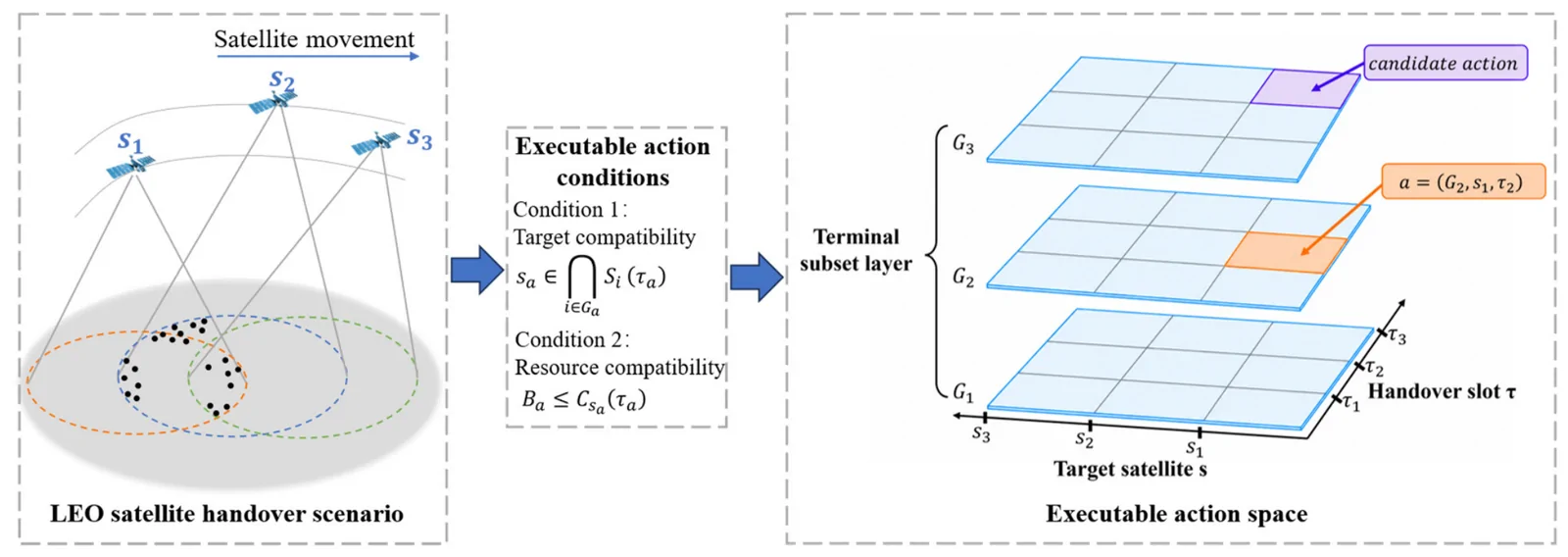
Illustration of executable group handover action construction.

Figure 1 illustrates the construction logic of executable group handover actions. In a LEO satellite handover scenario, terminals located in overlapping satellite coverage may have multiple feasible target satellites within a handover slot. A terminal subset, a target satellite, and a handover slot form an executable group handover action only when the target compatibility and resource compatibility conditions are satisfied.

For any action $a=\left(G_{a},s_{a},\tau_{a}\right)$, its executability is jointly determined by target compatibility and resource compatibility. First, target compatibility requires that the target satellite $s_{a}$ belongs to the candidate target satellite sets of all terminals in $G_{a}$ at slot $\tau_{a}$, which can be written as

(11) $$s_{a}\in \underset{i\in G_{a}}{⋂}S_{i}\left(\tau_{a}\right).$$

This condition ensures that all terminals in $G_{a}$ can be handed over to the same target satellite in the same slot. Second, resource compatibility requires that the total bandwidth demand of action a does not exceed the residual bandwidth available for handover access at satellite $s_{a}$ in slot $\tau_{a}$, which is expressed as

(12) $$B_{a}\leq C_{s_{a}}\left(\tau_{a}\right),$$

where $B_{a}$ denotes the total bandwidth demand of action a, defined as

(13) $$B_{a}=\sum_{i\in G_{a}}b_{i,s_{a}}\left(\tau_{a}\right).$$

Accordingly, the complete executable group handover action set is defined as

(14) $$A=\left\{a=\left(G_{a},s_{a},\tau_{a}\right)|G_{a}\subseteq U^{ho},\tau_{a}\in T,s_{a}\in \underset{i\in G_{a}}{⋂}S_{i}\left(\tau_{a}\right),B_{a}\leq C_{s_{a}}\left(\tau_{a}\right)\right\}.$$

It should be emphasized that $G_{a}$ is not a clustering result generated independently before optimization. Instead, $G_{a}$, $s_{a}$, and $\tau_{a}$ jointly define an executable group handover action. Therefore, the subsequent optimization does not select target satellites for fixed terminal groups, but directly selects an action combination from the executable action space A to improve the overall handover performance.

The executable action set A provides the decision space for group handover optimization. Based on this action-level representation, the following subsection further models the signaling overhead generated by executing group handover actions.

### 2.3. Group Handover Signaling Overhead Model

To quantify the control-plane overhead introduced by group handover, this paper establishes a field-level signaling overhead model for access-side handover control. The model focuses on the signaling exchanged between terminals and satellite access nodes, as well as that between the source and target access nodes during handover preparation and execution. Core-network-side signaling, such as path switching, session update, and context release, is beyond the scope of this model.

In the considered access-side handover process, the main control messages include measurement reporting, handover request, handover acknowledgement, reconfiguration, and completion feedback. Measurement reporting and completion feedback are terminal-level messages and are counted separately for each terminal. Handover request, handover acknowledgement, and reconfiguration messages contain action-level information shared by the terminals participating in the same group handover action. These messages can be aggregated in the group handover process.

The access-side messages considered above follow the standard single-UE handover procedure in 3GPP TS 38.331 and TS 38.423. For NTN, 3GPP TR 38.821 notes that a potentially very large number of UEs may need to perform handover at a given time, leading to possibly large signalling overhead. TR 38.821 further states that common signalling in the handover configuration can be broadcast, whereas the handover command is UE-specific and requires dedicated signalling. Accordingly, this paper abstracts the handover signalling model in terms of fields. When multiple terminals share the same target satellite and handover slot and form one executable action, the signalling is modeled with common fields and user-level fields.

**Figure 2:**
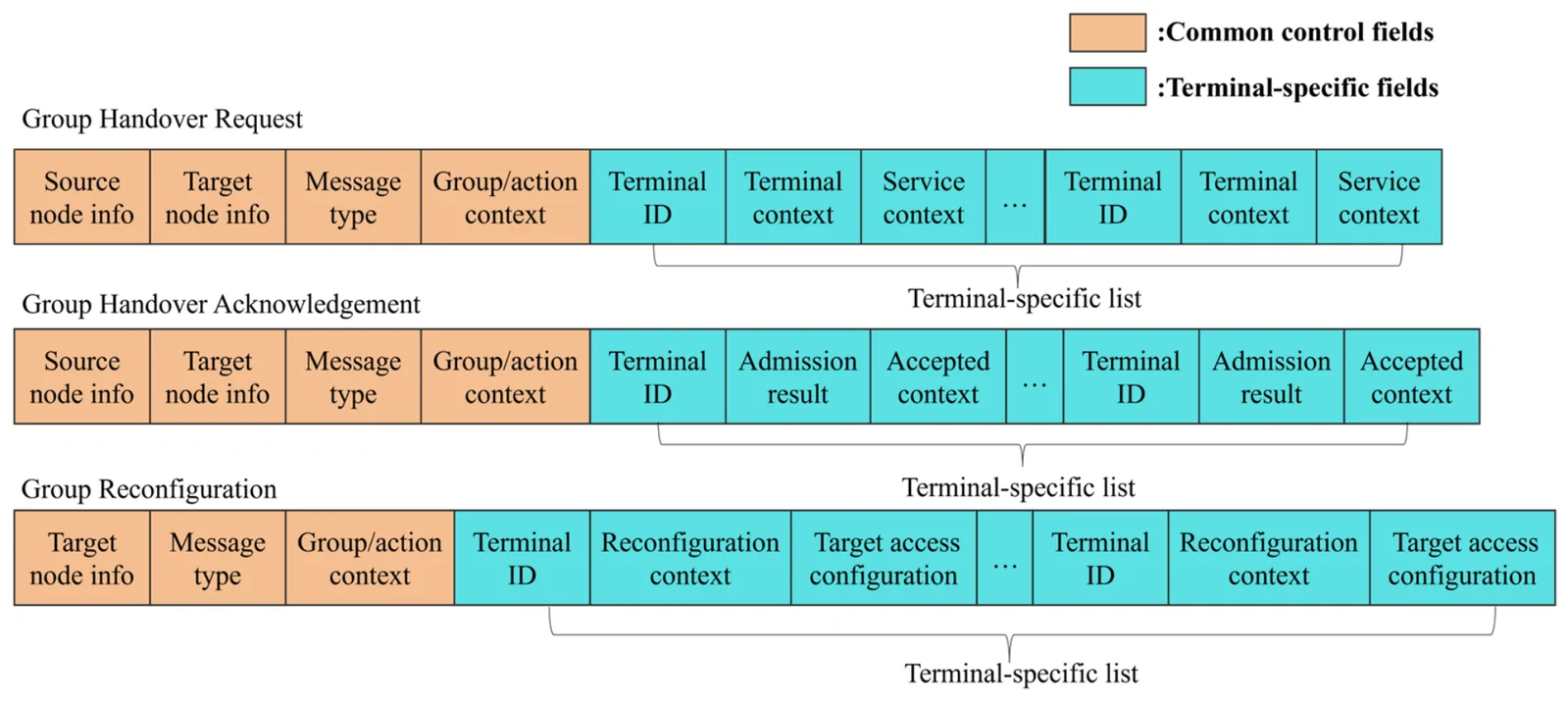
Signaling field structure for group handover.

Figure 2 illustrates the field-level structure of group handover signaling considered in this paper. As shown in Figure 2, the group handover request, group handover acknowledgement, and group reconfiguration messages are represented by a common part and a terminal-specific list. For the group handover request, the common part contains source node information, target node information, message type, and group/action context, while the terminal-specific list contains terminal ID, terminal context, and service context. For the group handover acknowledgement, the common part contains source node information, target node information, message type, and group/action context, while the terminal-specific list contains terminal ID, admission result, and accepted context. For the group reconfiguration message, the common part contains target node information, message type, and group/action context, while the terminal-specific list contains terminal ID, reconfiguration context, and target access configuration. The common part is transmitted once for a group handover action. The terminal-specific list refers to the collection of terminal-level field entries appended to the same group handover message.

Let $M_{agg}=\left\{req,ack,recfg\right\}$ denote the set of group handover signaling messages that can be aggregated, corresponding to the group handover request, group handover acknowledgement, and group reconfiguration messages, respectively. For each $m\in M_{agg}$, let $C_{m}^{com}$ denote the overhead of the common part, and let $C_{m}^{ter}$ denote the overhead of one terminal-specific field entry. For a group handover action $a=\left(G_{a},s_{a},\tau_{a}\right)$, the signaling overhead of message m is given by

(15) $$C_{m}\left(G_{a}\right)=C_{m}^{com}+\left|G_{a}\right|C_{m}^{ter},\;m\in M_{agg}.$$

For the conventional single-terminal handover scheme, each handover terminal independently triggers the access-side handover signaling procedure. Therefore, the signaling overhead associated with terminal i is expressed as

(16) $$C_{i}^{SU}=C_{meas}+C_{comp}+\sum_{m\in M_{agg}}\left(C_{m}^{com}+C_{m}^{ter}\right),$$

where $C_{meas}$ and $C_{comp}$ denote the measurement reporting overhead and completion feedback overhead of one terminal, respectively. The total signaling overhead of the single-terminal handover scheme is then given by

(17) $$C_{SU}=\sum_{i\in U^{ho}}C_{i}^{SU}.$$

For a group handover action $a=\left(G_{a},s_{a},\tau_{a}\right)$, the measurement reporting and completion feedback overheads are counted for each terminal in $G_{a}$, while the request, acknowledgement, and reconfiguration messages are aggregated according to (15). Therefore, the access-side signaling overhead of action a is given by

(18) $$C_{a}^{GH}=\left|G_{a}\right|\left(C_{meas}+C_{comp}\right)+\sum_{m\in M_{agg}}\left(C_{m}^{com}+\left|G_{a}\right|C_{m}^{ter}\right).$$

Let $A_{sel}\subseteq A$ denote the set of group handover actions triggered in the current decision window. The total signaling overhead of the group handover scheme is then expressed as

(19) $$C_{GH}=\sum_{a\in A_{sel}}C_{a}^{GH}.$$

### 2.4. Group Handover Optimization Problem

Based on the executable group handover action set defined above, the group handover decision in the LEO satellite network is formulated as an action selection optimization problem. Let A denote the set of all executable group handover actions within the current decision window. For each action a∈A, its terminal set, target satellite, execution slot, and bandwidth demand are denoted by $G_{a}$, $s_{a}$, $\tau_{a}$, and $B_{a}$, respectively. A binary decision variable $x_{a}$ is introduced to indicate whether action a is selected, where $x_{a}=1$ if action a is included in the final group handover scheme, and $x_{a}=0$ otherwise. To indicate whether a handover terminal fails to complete handover within the current decision window, a binary handover failure indicator $z_{i}$ is introduced. Specifically, $z_{i}=1$ if terminal i is not covered by any selected group handover action, indicating that its handover fails within the current window; otherwise, $z_{i}=0$. The group handover optimization problem can then be formulated as

(20) $$\begin{array}{cc} \min & \sum_{a\in A}c_{a}x_{a}+P\sum_{i\in U^{ho}}z_{i}\\ s.t. & \sum_{a\in A:i\in G_{a}}x_{a}+z_{i}=1,\;\forall i\in U^{ho},\\ & \sum_{a\in A:s_{a}=s,\tau_{a}=\tau }B_{a}x_{a}\leq C_{s}\left(\tau \right),\;\forall s\in S,\;\tau \in T,\\ & x_{a}\in \left\{0,1\right\},\;\forall a\in A,\\ & z_{i}\in \left\{0,1\right\},\;\forall i\in U^{ho}. \end{array}$$

where $c_{a}$ denotes the unit control cost of executing group handover action a. Accordingly, the first term in the objective function counts the selected group handover actions. P denotes the penalty for each failed handover terminal.

The signaling overhead in bytes is not directly included in the objective function and is evaluated separately using the field-level model in Section 2.3. With fixed field sizes and the same number of successfully handed-over terminals, reducing the number of handover actions reduces the overhead associated with repeated transmission of common fields.

The first constraint is the terminal coverage constraint, which ensures that each handover terminal is associated with at most one selected group handover action within the current decision window. If a terminal is not covered by any selected action, the corresponding handover failure indicator is set to 1. The second constraint is the satellite-slot capacity constraint, which ensures that the total bandwidth occupied by multiple group handover actions targeting the same satellite in the same handover slot does not exceed the residual bandwidth available for handover access at that satellite.

The above formulation takes executable group handover actions as the basic decision units, enabling terminal grouping, target satellite selection, and handover-slot determination to be jointly completed in a unified action space. Different from conventional group-then-handover methods, the proposed formulation does not fix terminal groups in advance, but directly selects group handover action combinations that are more beneficial to the overall handover performance from the executable action space.

### 2.5. Theoretical Analysis of Action-Space Loss Induced by Fixed Pre-Grouping

Conventional group handover methods typically follow a two-stage “grouping first and scheduling later” procedure: terminals are first clustered into fixed groups according to features such as location, channel state, or handover timing, after which a target satellite and a handover slot are selected for each fixed group. Although pre-grouping reduces the size of the subsequent scheduling problem, it also restricts in advance the terminal combinations available in the scheduling stage. This subsection analyzes, from a mathematical perspective, the resulting restriction on subsequent decisions and the associated performance loss, and clarifies which feasible actions are excluded by pre-grouping. First, the complete executable-action space and the fixed pre-grouping action space are defined, and the relationship between the two spaces is examined. Second, three classes of executable actions excluded by fixed pre-grouping are characterized. Finally, the performance loss induced by pre-grouping is analyzed.

#### 2.5.1. Relationship Between the Complete and Pre-Grouping Action Spaces

Let Q=S×T denote the set of satellite–slot resource units, where $q=\left(s,\tau \right)$. Following the executable-action definition introduced earlier, a group handover action is represented as $a=\left(G_{a},s_{a},\tau_{a}\right)$, where $G_{a}\subseteq U^{ho}$ is the set of terminals participating in action a, while $s_{a}$ and $\tau_{a}$ are its target satellite and execution slot, respectively.

For a given satellite–slot $q=\left(s,\tau \right)$, the family of nonempty terminal sets that can form executable actions on this resource unit is defined as

(21) $$F_{q}=\left\{G\subseteq U^{ho}:G\neq \emptyset ,s\in {⋂}_{i\in G}S_{i}\left(\tau \right),\sum_{i\in G}b_{i,s}\left(\tau \right)\leq C_{s}\left(\tau \right)\right\},$$

where $S_{i}\left(\tau \right)$ is the candidate-satellite set of terminal i in slot τ, $b_{i,s}\left(\tau \right)$ is the bandwidth required by terminal i to access satellite s, and $C_{s}\left(\tau \right)$ is the available access capacity of satellite s in slot τ. Accordingly, the complete executable-action space is

(22) $$A={⋃}_{q=\left(s,\tau \right)\in Q}\left\{\left(G,s,\tau \right):G\in F_{q}\right\}.$$

Suppose that a conventional pre-grouping method generates K mutually disjoint fixed terminal groups before scheduling. The collection of these pre-grouped terminal groups is denoted by $\Pi =\left\{H_{1},H_{2},\ldots ,H_{K}\right\}$, where $H_{k}\cap H_{l}=\emptyset$ for k≠l and ${⋃}_{k=1}^{K}H_{k}=U^{ho}$. If the subsequent scheduling stage can only treat each complete group $H_{k}$ as an unsplittable handover unit, the resulting fixed pre-grouping action space is

(23) $$A_{\Pi }=\left\{\left(H_{k},s,\tau \right)\in A:H_{k}\in \Pi \right\}.$$

According to (22) and (23), every fixed pre-grouping action belongs to the complete executable-action space. Therefore,

(24) $$A_{\Pi }\subseteq A.$$

#### 2.5.2. Executable Actions Excluded by Fixed Pre-Grouping

Define the set of executable actions excluded by fixed pre-grouping as $L_{\Pi }=A\setminus A_{\Pi }$. Here, $L_{\Pi }$ contains actions that satisfy the executability conditions in (21) but cannot be used by fixed pre-grouping methods. The action-space loss caused by fixed pre-grouping can be divided into the following three categories.

(1) Intra-group splitting actions. For a fixed terminal group $H_{k}\in \Pi$, the complete action space allows any nonempty proper subset $G_{a}$ to form an action independently, where $\emptyset ⊊G_{a}⊊H_{k}$.

(2) Cross-group regrouping actions. The complete action space allows terminals originally assigned to different fixed groups to jointly form an action. An action is called a cross-group regrouping action if it spans at least two fixed groups and splits at least one of them. Specifically, $G_{a}\cap H_{k}\neq \emptyset$, $G_{a}\cap H_{l}\neq \emptyset$, and k≠l, while at least one fixed group satisfies $\emptyset ⊊G_{a}\cap H_{k}⊊H_{k}$.

(3) Fixed-group merging actions. The complete action space allows two or more complete fixed terminal groups to be merged into a new action, where $G_{a}=H_{k_{1}}\cup H_{k_{2}}\cup \ldots \cup H_{k_{r}}$ and r≥2.

For any of the above three terminal-set structures, if $G_{a}$ satisfies the executability conditions in (21), the corresponding action $a=\left(G_{a},s_{a},\tau_{a}\right)$ belongs to the complete action space A but not to the fixed pre-grouping action space $A_{\Pi }$.

#### 2.5.3. Performance Loss Induced by Fixed Pre-Grouping

To analyze the performance loss caused by fixed pre-grouping, the action space A in the original problem is replaced by $A_{\Pi }$, while the objective function and all other constraints remain unchanged. This yields the fixed pre-grouping restricted master problem.

Consider the LP relaxation of this restricted master problem. Let $\pi_{i}$ denote the dual variable associated with the coverage constraint of terminal i, and let $\mu_{s,\tau }$ denote the dual variable associated with the capacity constraint of satellite s in slot τ. For any executable action $a=\left(G_{a},s_{a},\tau_{a}\right)\in A$, its reduced cost is evaluated using the optimal dual solution of this LP:

(25) $${\bar{c}}_{a}=c_{a}-\sum_{i\in G_{a}}\pi_{i}+\mu_{s_{a},\tau_{a}}B_{a}.$$

Here, $c_{a}$ is the action execution cost, $\sum_{i\in G_{a}}\pi_{i}$ is the dual benefit obtained by covering the terminals in the action, and $\mu_{s_{a},\tau_{a}}B_{a}$ is the dual resource cost associated with its occupation of satellite–slot capacity. The net improvement value of action a is defined as

(26) $$R\left(a\right)=\sum_{i\in G_{a}}\pi_{i}-\mu_{s_{a},\tau_{a}}B_{a}-c_{a}=-{\bar{c}}_{a}.$$

Thus, $R\left(a\right)>0$ is equivalent to ${\bar{c}}_{a}<$ 0. A negative reduced cost indicates that the dual benefit generated by covering the terminals in action a exceeds its action execution cost and resource-occupation cost, implying that action a has the potential to improve the current LP solution.

At the optimum of the fixed pre-grouping LP, all permitted actions satisfy ${\bar{c}}_{a}\geq 0$ for $a\in A_{\Pi }$. However, an excluded action $a\in L_{\Pi }$ is absent from the restricted master problem, and its corresponding constraint is therefore not included in the dual problem. Consequently, its reduced cost may still be negative.

To characterize the maximum improvement potential omitted by fixed pre-grouping, define

(27) $$\delta_{\Pi }=\min\left\{0,\underset{a\in L_{\Pi }}{\min}{\bar{c}}_{a}\right\}.$$

If $\delta_{\Pi }=0$, none of the excluded actions has a negative reduced cost. If $\delta_{\Pi }<0$, at least one executable action with improvement potential has been excluded by fixed pre-grouping.

Let $v_{\Pi }$ and v denote the optimal LP-relaxation objective values of the fixed pre-grouping restricted master problem and the complete-action master problem, respectively. Since $A_{\Pi }\subseteq A$, the complete-action problem includes every feasible solution of the restricted problem, and hence $v\leq v_{\Pi }.$

**Proposition 1.** 
*The LP objective loss caused by fixed pre-grouping satisfies.*

(28) $$0\leq v_{\Pi }-v\leq -N\delta_{\Pi }.$$

**Proof of Proposition 1.**  Let $\left(\hat{\pi },\hat{\mu }\right)$ be an optimal dual solution of the fixed pre-grouping LP. The capacity constraints imply ${\hat{\mu }}_{s,\tau }\geq 0$. The corresponding optimal dual objective value equals $v_{\Pi }$:

(29) $$v_{\Pi }=\sum_{i\in U^{ho}}{\hat{\pi }}_{i}-\sum_{\left(s,\tau \right)\in Q}{\hat{\mu }}_{s,\tau }C_{s}\left(\tau \right).$$

Let $\left(x^{*},z^{*}\right)$ be an optimal solution of the complete-action LP. Define the residual capacity of satellite s in slot τ as

(30) $$r_{s,\tau }^{*}=C_{s}\left(\tau \right)-\sum_{\begin{array}{c} a\in A:\\ s_{a}=s,\;\tau_{a}=\tau \end{array}}B_{a}x_{a}^{*}\geq 0.$$

According to the reduced-cost definition in (25),

(31) $$c_{a}=\sum_{i\in G_{a}}{\hat{\pi }}_{i}-{\hat{\mu }}_{s_{a},\tau_{a}}B_{a}+{\bar{c}}_{a}.$$

The reduced cost of the failure variable $z_{i}$ is ${\bar{c}}_{i}^{z}=P-{\hat{\pi }}_{i}\geq 0.$ Substituting (31) into the complete-action LP objective and using the terminal-coverage constraint $\sum_{a:i\in G_{a}}x_{a}^{*}+z_{i}^{*}=1$, together with (29) and (30), yields

(32) $$v=v_{\Pi }+\sum_{\left(s,\tau \right)\in Q}{\hat{\mu }}_{s,\tau }r_{s,\tau }^{*}+\sum_{a\in A}{\bar{c}}_{a}x_{a}^{*}+\sum_{i\in U^{ho}}{\bar{c}}_{i}^{z}z_{i}^{*}.$$

Because ${\hat{\mu }}_{s,\tau }r_{s,\tau }^{*}\geq 0,{\bar{c}}_{i}^{z}z_{i}^{*}\geq 0$, and ${\bar{c}}_{a}\geq 0$ for all $a\in A_{\Pi }$, negative contributions in (32) can only arise from excluded actions. Therefore,

(33) $$v\geq v_{\Pi }+\sum_{a\in L_{\Pi }}{\bar{c}}_{a}x_{a}^{*}\geq v_{\Pi }+\delta_{\Pi }\sum_{a\in L_{\Pi }}x_{a}^{*}.$$

Summing all terminal-coverage constraints gives

(34) $$\sum_{a\in A}\left|G_{a}\right|x_{a}^{*}+\sum_{i\in U^{ho}}z_{i}^{*}=N.$$

Since every action covers at least one terminal, $\left|G_{a}\right|\geq 1$, and hence

(35) $$\sum_{a\in L_{\Pi }}x_{a}^{*}\leq \sum_{a\in A}x_{a}^{*}\leq N.$$

Because $\delta_{\Pi }\leq 0$, (35) implies

(36) $$\delta_{\Pi }\sum_{a\in L_{\Pi }}x_{a}^{*}\geq N\delta_{\Pi }.$$

Combining (33) and (36) gives $v\geq v_{\Pi }+N\delta_{\Pi }.$ Since $v\leq v_{\Pi }$, it follows that $0\leq v_{\Pi }-v\leq -N\delta_{\Pi }$, which proves (28). □

If $\delta_{\Pi }=0,$ then (28) gives $v=v_{\Pi }$, indicating no LP objective loss due to fixed pre-grouping. If $\delta_{\Pi }<0$, then $-N\delta_{\Pi }$ provides an upper bound on the LP objective loss $v_{\Pi }-v$.

Since the final handover scheme must satisfy the binary constraints, the LP loss bound in Proposition 1 only characterizes the effect of action-space restriction at the LP-relaxation level and cannot directly determine whether the final integer solution is improved. Therefore, the integer-solution performance of the complete-action model and the fixed pre-grouping model is further compared below.

Let X and $X_{\Pi }$ denote the integer feasible sets of the complete-action model and the fixed pre-grouping model, respectively, and let $J^{*}$ and $J_{\Pi }^{*}$ denote their optimal objective values.

**Proposition 2.** 
*The complete-action model and the fixed pre-grouping model satisfy.*

(37) $$X_{\Pi }\subseteq X,\;J^{*}\leq J_{\Pi }^{*}.$$

Let c denote the common action cost. Suppose that the optimal fixed pre-grouping solution uses r actions, while an integer feasible solution in the complete action space uses $r^{\prime }$ actions and reduces the number of failed terminals by w. If the benefit of recovering these w failed terminals exceeds the resulting increase in action cost, namely, $Pw>\left(r^{\prime }-r\right)c$, then the complete action space admits a strictly better handover solution than fixed pre-grouping, which yields

(38) $$J^{*}<J_{\Pi }^{*}.$$

**Proof of Proposition 2.**  Consider any integer feasible solution $\left(x^{\Pi },z^{\Pi }\right)$ of the fixed pre-grouping model. In the complete-action model, retain the values of all pre-grouping action variables and failure variables, and set the variables associated with all actions in $L_{\Pi }$ to zero.

Since $A_{\Pi }\subseteq$ A, every fixed pre-grouping action is also executable in the complete-action model. This construction therefore leaves the terminal coverage, satellite–slot resource occupation, and objective value unchanged. Hence, every integer feasible solution of the fixed pre-grouping model corresponds to an integer feasible solution of the complete-action model with the same objective value. Consequently, $X_{\Pi }\subseteq X$ and $J^{*}\leq J_{\Pi }^{*}$.

For the strict-improvement condition, the fixed pre-grouping optimum uses r actions, whereas the integer feasible solution in the complete action space uses $r^{\prime }$ actions and reduces the number of failed terminals by w. The difference between their objective values is

(39) $$\Delta J=\left(r^{\prime }-r\right)c-Pw,$$

where $\left(r^{\prime }-r\right)c$ is the change in action cost and Pw is the reduction in failure penalty obtained by recovering w failed terminals.

If $P_{w}>\left(r^{\prime }-r\right)c$, then ΔJ<0, indicating that the complete action space contains an integer feasible solution whose objective value is strictly lower than $J_{\Pi }^{*}$. Therefore, the optimal objective value of the complete-action model satisfies $J^{*}<J_{\Pi }^{*}$. This completes the proof. □

## 3. Proposed GHACG Algorithm

The executable action modeling developed in Section 2 treats the set of terminals involved in a group handover action $G_{a}$, the target satellite $s_{a}$, and the handover slot $\tau_{a}$ as an integrated group handover decision unit. This enables the algorithm to search directly in the action space jointly formed by these factors, rather than first fixing terminal groups and then determining the target satellite and handover slot for each group. Such an action-level representation avoids the action-space loss caused by conventional pre-grouping methods, allowing the algorithm to generate and select high-quality group handover actions from a more complete executable action space.

However, since each group handover action involves the joint selection of participating terminals, a target satellite, and a handover slot, the complete executable action space grows rapidly with the number of handover terminals. Therefore, the algorithm needs to select group handover actions satisfying target-satellite compatibility and resource compatibility from a large number of candidate actions, and further form an action combination with good overall performance. In large-scale concurrent handover scenarios, how to efficiently search for high-quality action combinations from the large executable action space becomes the key challenge of algorithm design.

To address this issue, this paper adopts the column generation principle to construct the solution framework. The core idea is to start from a restricted executable action set and iteratively generate executable group handover action columns with potential improvement value during the solution process. Since each group handover action corresponds to a decision variable in the master problem, column generation naturally couples action generation with action selection. By using the dual information of the current restricted master problem to identify action columns with negative reduced cost, the algorithm preferentially expands the candidate action set with actions that are more likely to improve the objective value, thereby reducing the difficulty of solving large-scale instances.

Under the column generation framework, this paper introduces dual-price-guided heuristic pricing and proposes a Group Handover Action Column Generation (GHACG) algorithm. The proposed algorithm decomposes the pricing problem according to the satellite-slot structure and uses the dual prices of the restricted master problem to generate high-value executable action columns. Since the linear relaxation of the restricted master problem may yield fractional action variables, an integer action recovery procedure with residual feasible action repair is designed to obtain the final executable group handover scheme.

### 3.1. Column Generation Framework

Since each decision variable $x_{a}$ in the complete optimization model corresponds to an executable group handover action a, an executable group handover action can be regarded as a column in the master problem under the column generation framework. As the size of the complete action set A grows rapidly with the number of handover terminals, directly solving the master problem over A is computationally intractable. Therefore, a restricted action set $A_{R}\subseteq A$ is introduced, and the restricted master problem is formulated over $A_{R}$.

To avoid fixing terminal groups before optimization, the algorithm starts from an action pool without pre-defined multi-terminal groups and then generates group handover actions through column generation. Therefore, in the initialization stage, the restricted action set is initialized with single-terminal executable handover actions. Specifically, if terminal i can hand over to satellite s in slot τ, a single-terminal handover action is constructed as $a=\left(\left\{i\right\},s,\tau \right).$ The constructed single-terminal handover action is added to the restricted master problem as an initial column. Accordingly, the initial restricted action set can be written as

(40) $$A_{R}^{0}=\left\{a=\left(\left\{i\right\},s,\tau \right)|i\in U^{ho},s\in S_{i}\left(\tau \right),\tau \in T,B_{a}=b_{i,s}\left(\tau \right)\leq C_{s}\left(\tau \right)\right\}.$$

In the subsequent column generation process, new executable group handover actions are generated by solving the pricing problem according to the optimization result of the current restricted master problem. Let $A_{new}^{k}$ denote the set of newly generated actions in iteration k. Then, the restricted action set is updated as

(41) $$A_{R}^{k+1}=A_{R}^{k}\cup A_{new}^{k}.$$

Through this reformulation, the complete action selection problem is transformed into an iterative solution process over restricted action sets. The restricted action set is initialized by single-terminal actions and is gradually expanded during the column generation process.

### 3.2. Restricted Master Problem and Pricing Subproblem

At the k-th column generation iteration, the restricted master problem is solved only over the current restricted action set $A_{R}^{k}$. The restricted master problem has the same objective function and constraint structure as the complete master problem, except that its decision variables correspond only to the actions in $A_{R}^{k}$. Therefore, the restricted master problem at iteration k can be directly obtained from the complete optimization model in Section 2 by replacing the complete action set A with the current restricted action set $A_{R}^{k}$.

In the column generation process, the pricing problem is used to search for executable actions outside the current restricted action set that can improve the objective value. To obtain the dual prices required for pricing, the restricted master problem is solved in its linear relaxation form. Let $\pi_{i}$ denote the dual variable associated with the terminal coverage constraint, and let $\mu_{s,\tau }$ denote the dual variable associated with the satellite-slot capacity constraint, where $\pi_{i}$ reflects the benefit of covering handover terminal i, and $\mu_{s,\tau }$ reflects the cost of occupying access bandwidth at satellite s in slot τ. Then, for any executable action $a\notin A_{R}^{k}$, its reduced cost can be written as

(42) $${\bar{c}}_{a}=c_{a}-\sum_{i\in G_{a}}\pi_{i}+\mu_{s_{a},\tau_{a}}B_{a}.$$

If there exists an action with ${\bar{c}}_{a}<0$, adding this action to the restricted master problem may further reduce the current linear relaxation objective value. Therefore, such an action is added to the restricted action set as a new column.

For a given satellite s and slot τ, the candidate terminal set that can participate in handover to this satellite-slot pair is defined as

(43) $$U_{s,\tau }=\left\{i\in U^{ho}|s\in S_{i}\left(\tau \right)\right\}.$$

Based on this candidate terminal set, the pricing subproblem can be formulated as

(44) $$\begin{array}{cc} \underset{G\subseteq U_{s,\tau }}{\min} & c_{\left(G,s,\tau \right)}-\sum_{i\in G}\pi_{i}+\mu_{s,\tau }\sum_{i\in G}b_{i,s}\left(\tau \right)\\ s.t. & \sum_{i\in G}b_{i,s}\left(\tau \right)\leq C_{s}\left(\tau \right). \end{array}$$

where $b_{i,s}\left(\tau \right)$ denotes the bandwidth required by terminal i when accessing satellite s in slot τ, and $c_{\left(G,s,\tau \right)}$ denotes the cost of the group handover action formed by terminal set G, target satellite s, and handover slot τ. If a given satellite-slot pair $\left(s,\tau \right)$ generates a terminal set G with negative reduced cost, the corresponding action $a=\left(G,s,\tau \right)$ is added to the new action set $A_{new}^{k}$.

### 3.3. Heuristic Pricing for Action Column Generation

The pricing subproblem needs to select a terminal subset G from the candidate terminal set $U_{s,\tau }$ for a given satellite-slot combination $\left(s,\tau \right)$, where s denotes the target satellite and τ denotes the handover slot. The objective is to construct an executable group handover action with a reduced cost as small as possible. Since the number of candidate terminal subsets increases rapidly with the number of terminals, directly searching all possible terminal combinations is computationally prohibitive in large-scale concurrent handover scenarios. Therefore, this paper designs a satellite-slot-oriented heuristic pricing method to quickly generate executable group handover actions with improvement value.

According to the reduced cost expression, for a fixed satellite-slot combination $\left(s,\tau \right)$, the individual reduced-cost gain of terminal i is defined as

(45) $$g_{i,s,\tau }=\pi_{i}-\mu_{s,\tau }b_{i,s}\left(\tau \right).$$

Here, $\pi_{i}$ represents the dual benefit of covering terminal i, while $\mu_{s,\tau }b_{i,s}\left(\tau \right)$ represents the dual resource cost caused by the bandwidth occupied by terminal i. A larger $g_{i,s,\tau }$ indicates that terminal i is more likely to reduce the reduced cost when included in an action associated with $\left(s,\tau \right)$.

In the heuristic pricing process, the algorithm first calculates $g_{i,s,\tau }$ for each candidate terminal in $U_{s,\tau }$, and sorts the candidate terminals in descending order of $g_{i,s,\tau }$. Then, under the residual bandwidth constraint $C_{s}\left(\tau \right)$, terminals with higher priority are sequentially added to the current candidate set to construct a candidate group handover action $a=\left(G,s,\tau \right)$. The generated candidate action is further evaluated according to its overall reduced cost. If ${\bar{c}}_{a}<0$, the action is added to the new action set $A_{new}^{k}$. If $A_{new}^{k}=\emptyset$, no executable action with negative reduced cost is generated by the heuristic pricing procedure in the current iteration, and the column generation process terminates. The process also terminates if the LP relaxation objective and the recovered integer objective do not improve for three consecutive iterations. Otherwise, the restricted action set is updated by $A_{R}^{k+1}=A_{R}^{k}\cup A_{new}^{k}$, and the next iteration starts.

For a given satellite-slot pair $\left(s,\tau \right)$, consider gain-ordered greedy construction over the complete candidate terminal set $U_{s,\tau }$, with $c_{a}=1$. If all candidate terminals have identical bandwidth requirements, the bandwidth constraint is equivalent to an upper bound on the number of selected terminals. For any given feasible subset size, replacing a selected terminal with an unselected terminal having a larger $g_{i,s,\tau }$ increases the total reduced-cost gain without changing bandwidth consumption. Moreover, adding another terminal with $g_{i,s,\tau }>0$ increases the total gain whenever capacity permits. Therefore, selecting terminals with $g_{i,s,\tau }>0$ in descending order of $g_{i,s,\tau }$, until no further such terminal can be accommodated or all have been selected, maximizes the total reduced-cost gain.

The same conclusion holds for heterogeneous bandwidth requirements when the residual bandwidth of target satellite s in slot τ can accommodate all candidate terminals with $g_{i,s,\tau }>0$. In this case, selecting all such terminals maximizes the total reduced-cost gain. Whenever a nonempty subset is obtained under either of these conditions, it minimizes the reduced cost of the corresponding pricing subproblem.

When candidate terminals have different bandwidth requirements and the residual bandwidth $C_{s}\left(\tau \right)$ of target satellite s in slot τ cannot accommodate all candidate terminals with $g_{i,s,\tau }>0$, subset construction involves a trade-off between individual reduced-cost gain and bandwidth consumption. Selecting a terminal with a large $g_{i,s,\tau }$ may leave insufficient bandwidth to accommodate a combination of other terminals whose total gain exceeds that of the selected terminal. Selecting terminals in descending order of $g_{i,s,\tau }$ does not guarantee the maximum total reduced-cost gain or the minimum action reduced cost. Consequently, some improving actions may remain undiscovered, potentially resulting in a loss of solution quality. To balance solution quality and computational efficiency, the heuristic constructs candidate actions by prioritizing promising terminals rather than enumerating all feasible terminal combinations, aiming to reduce the computational burden of action generation in large-scale handover scenarios.

Based on the above restricted master problem and heuristic pricing procedure, the overall GHACG algorithm is summarized in Algorithm 1.
**Algorithm 1.** Group Handover Action Column Generation**Input:** $U^{ho},S,T,S_{i}(\tau ),b_{i,s}(\tau ),C_{s}(\tau ),c_{a},P,\varepsilon .$ **Output:** $A_{sel}.$1.Initialize k=0 
$\mathrm{and}\;A_{R}^{0}=\emptyset .$
2.**for each** 
$i\in U^{ho},$
τ∈T,
$\;\mathrm{and}\;s\in S_{i}(\tau )$ 
**do**
3.  Construct and add the single-terminal action a=({i},s,τ) to $A_{R}^{0}.$4.**end for**5.**while true do**6.  Solve the LP relaxation of the restricted master problem over
$A_{R}^{k},$ and obtain the dual prices $\pi_{i}$ and
$\mu_{s,\tau }.$
7.  
$\mathrm{Set}\;A_{new}^{k}=\emptyset .$
8.  
for each s∈S 
and τ∈T
**do**
9.      Construct $U_{s,\tau }=\{i\in U^{ho}|s\in S_{i}(\tau )\}.$
10.     
$\mathrm{if}\;U_{s,\tau }\neq \emptyset$
**then**
11.        Compute $g_{i,s,\tau }=\pi_{i}-\mu_{s,\tau }b_{i,s}(\tau )$
$\mathrm{for}\;\mathrm{all}\;i\in U_{s,\tau }.$
12.        Sort terminals in
$U_{s,\tau }$ in descending order of
$g_{i,s,\tau }.$
13.        Generate feasible candidate subset(s)
G under
$\sum_{i\in G}b_{i,s}(\tau )\leq C_{s}(\tau ).$
14.        **for each** generated nonempty subset
G
**do**
15.          Form a=(G,s,τ) and compute ${\bar{c}}_{a}.$
16.          
$\;\mathrm{if}\;a\notin A_{R}^{k}$ and
${\bar{c}}_{a}<-\varepsilon$
**then**
17.             Add a to $A_{new}^{k}.$
18.          
**end if**
19.        
**end for**
20.     
**end if**
21.  
**end for**
22.  
$\mathrm{if}\;A_{new}^{k}=\emptyset$
**then**
23.     **break**.24.  
**end if**
25.  **if** the LP objective and the integer objective have not improved for three consecutive iterations **then**26.  
**break.**
27.  
**end if**
28.  
$A_{R}^{k+1}=A_{R}^{k}\cup A_{new}^{k}.$
29.  
k=k+1.
30.**end while**31.$\mathrm{Obtain}\;A_{sel}$ by applying Algorithm 2$\;\mathrm{to}\;\mathrm{the}\;\mathrm{final}\;\mathrm{restricted}\;\mathrm{action}\;\mathrm{set}\;A_{R}^{k}.$32.**return** $A_{sel}.$

### 3.4. Integer Action Recovery

After the column generation process terminates, the final restricted action set contains a collection of executable group handover actions generated during pricing. However, the solution of the relaxed restricted master problem may contain fractional variables and therefore cannot be directly implemented as a handover schedule. To obtain an executable integer solution, this paper performs integer action recovery over the final restricted action set. Specifically, the recovery procedure progressively selects complete group handover actions from the final restricted action set according to their residual feasibility and residual benefit. In this way, fractional action variables are replaced by a set of selected executable actions that satisfies terminal coverage and satellite-slot capacity constraints.

During the integer recovery procedure, executable group handover actions are progressively selected from the final restricted action set $A_{R}$. Let $A_{sel}^{t}$ denote the set of actions selected before step t. Then, the set of terminals that remain uncovered at step t is given by

(46) $$U_{res}^{t}=U^{ho}\setminus \underset{a\in A_{sel}^{t}}{⋃}G_{a}.$$

The residual bandwidth resource of satellite s in slot τ is updated as

(47) $$C_{res}^{t}\left(s,\tau \right)=C_{s}\left(\tau \right)-\sum_{a\in A_{sel}^{t}:\;s_{a}=s,\;\tau_{a}=\tau }B_{a}.$$

Based on the remaining uncovered terminals and residual bandwidth resources, the residual feasible action set at step t is defined as

(48) $$A_{res}^{t}=\left\{a\in A_{R}\setminus A_{sel}^{t}|G_{a}\subseteq U_{res}^{t},B_{a}\leq C_{res}^{t}\left(s_{a},\tau_{a}\right)\right\}.$$

At recovery step t, the algorithm evaluates the residual benefit of each action in the current residual feasible action set $A_{res}^{t}$. For any action $a\in A_{res}^{t}$, its residual benefit is defined as

(49) $$\Delta_{a}^{t}=P\left|G_{a}\right|-c_{a}.$$

Here, $P\left|G_{a}\right|$ represents the reduction in handover failure penalty obtained by covering the remaining terminals in $G_{a}$, while $c_{a}$ denotes the control cost of executing action a. Therefore, at recovery step t, the action with the maximum residual benefit is selected as

(50) $$a^{t}=\arg\underset{a\in A_{\mathrm{res}}^{t}}{\max}\Delta_{a}^{t}.$$

If $\Delta_{a^{t}}^{t}>0$, action $a^{t}$ is added to the selected action set, and the selected action set is updated as

(51) $$A_{sel}^{t+1}=A_{sel}^{t}\cup \left\{a^{t}\right\}.$$

The algorithm then proceeds to the next recovery step. If $A_{res}^{t}=\emptyset$ or $\Delta_{a^{t}}^{t}\leq 0$, the integer recovery procedure terminates. The final selected action set $A_{sel}$ is returned as the executable integer group handover scheme.

The complete integer action recovery procedure is summarized in Algorithm 2.
**Algorithm 2.** Integer Action Recovery with Residual Feasible Repair**Inputs:** $A_{R},U^{ho},S,T,C_{s}(\tau ),B_{a},c_{a},P.$ **Output:** 
$A_{sel}.$
1.$\mathrm{Initialize}\;A_{sel}^{0}=\emptyset$ 
and t=0.
2.**while true do**3.  $U_{res}^{t}=U^{ho}\setminus {⋃}_{a\in A_{sel}^{t}}G_{a}.$
4.  **for each** 
s∈S 
and τ∈T
**do**
5.    $C_{res}^{t}(s,\tau )=C_{s}(\tau )-\sum_{a\in A_{sel}^{t}:s_{a}=s,\tau_{a}=\tau }B_{a}.$
6.  **end for**
7.  $A_{res}^{t}=\{a\in A_{R}\setminus A_{sel}^{t}|G_{a}\subseteq U_{res}^{t},B_{a}\leq C_{res}^{t}(s_{a},\tau_{a})\}.$
8.  **if** 
$A_{res}^{t}=\emptyset$ 
**then**
9.    **break**.10.  **end if**
11.  **for each** 
$a\in A_{res}^{t}$ 
**do**
12.    $\Delta_{a}^{t}=P|G_{a}|-c_{a}.$
13.  **end for**
14.  Select $a^{t}\in \arg\;\max_{a\in A_{res}^{t}}\Delta_{a}^{t}.$
15.  **if** 
$\Delta_{a^{t}}^{t}\leq 0$ 
**then**
16.    **break**.17.  **end if**
18.  $A_{sel}^{t+1}=A_{sel}^{t}\cup \{a^{t}\}.$
19.  t=t+1.
20.**end while**21.$A_{sel}=A_{sel}^{t}.$22.**return** 
$A_{sel}.$


## 4. Simulation Results and Analysis

### 4.1. Simulation Setup

To evaluate the effectiveness of the proposed GHACG algorithm in large-scale LEO satellite group handover scenarios, a system-level simulation environment is established. The satellite constellation is configured by referring to Starlink Gen1 Shell 1, and the satellite orbital data used in the simulations are provided by ephemeris files generated from STK. The constellation consists of 1584 LEO satellites distributed over 72 orbital planes, with 22 satellites in each plane. The orbital altitude and inclination are set to 550 km and 53°, respectively.

The ground terminals are distributed in a wide-area region in northwestern China, defined by the latitude range $\left[36^{\circ }\;N,46^{\circ }\;N\right]$ and the longitude range $\left[86^{\circ }\;E,106^{\circ }\;E\right]$. The ground terminal locations follow a binomial point process (BPP), where terminals are independently and uniformly distributed within the considered region. This paper treats the terminal population as a controlled experimental variable and focuses on comparing handover performance across terminal scales. The service rate requirement of each terminal is selected from $\left\{2,5,10\right\}$ Mbps with probabilities 0.4, 0.4, and 0.2, respectively.

The link quality is evaluated using the link model described in Section 2.1. The main simulations consider an outdoor LOS and clear-sky scenario, in which free-space path loss, shadow fading, atmospheric gas attenuation, and tropospheric scintillation are included, whereas clutter loss, building-entry loss, rain attenuation, and cloud attenuation are set to zero. Co-channel interference is neglected in the nominal scenario and is examined separately in the robustness experiments. The carrier frequency is set to 20 GHz, and the minimum elevation angle is set to 30°. The minimum remaining service time is set to $T_{min}^{rem}=180\;s$. Following the LEO Ka-band calibration parameters in 3GPP TR 38.821 [9], the satellite EIRP is set to 30 dBW, corresponding to an EIRP density of 4 dBW/MHz over a channel bandwidth of 400 MHz. At 20 GHz, the zenith atmospheric attenuation is set to 0.26 dB. For each terminal–satellite link, the shadow-fading standard deviation and tropospheric scintillation loss are selected according to the nearest reference elevation angle specified in the 3GPP NTN channel model [7]. The link SINR, achievable spectral efficiency, and equivalent bandwidth demand are calculated according to the models in Section 2. The residual bandwidth resource available for handover access at satellite s in slot τ, denoted by $C_{s}\left(\tau \right)$, is set to 1500 MHz. It should be noted that $C_{s}\left(\tau \right)$ represents the bandwidth resource available for admission in the current decision slot, rather than the total system bandwidth of the satellite.

The satellite ephemeris generated by STK is sampled with a time resolution of 1 s. Each simulation run covers 20 min and consists of 40 decision slots, each with a duration of 30 s. In each decision slot, the system updates satellite positions, terminal-satellite visibility relationships, link quality, and candidate target satellite sets according to the ephemeris data, and then makes handover decisions based on the updated network state. To reduce the impact of the randomness in terminal deployment and service rate requirements on the simulation results, each of the six terminal population settings is evaluated under five independent random seeds, and the averaged results are reported. Handover demand does not occur in every slot. In the selected ephemeris window, 6 of the 40 slots contain handover demand. This count is the same for all six terminal scales. The main simulation parameters are summarized in Table 2.

Based on the above simulation settings, the proposed GHACG algorithm is compared with four baseline schemes, including the conventional single-user handover scheme (SG) and three representative group handover schemes from published studies, namely massive-user group handover (MGH), double-grouping-based group handover (DGGH), and multi-layer-graph-based group handover (MLG) [41,42,46]. SG decides for each terminal independently and greedily chooses a currently feasible target satellite and slot under the residual bandwidth. MGH first applies hierarchical clustering and then selects target satellites by a division-restricted network-flow model, scheduling each clustered group as a whole [41]. DGGH first forms spatial user groups by hierarchical clustering and then performs satellite grouping to select common target satellites [42]. MLG first clusters devices with a CF-tree and then matches groups, target satellites, and handover slots on a multi-layer graph by minimum-cost maximum-flow [46]. For a fair comparison, all schemes use the same satellite constellation, terminal distribution, link-budget parameters, and feasible target satellite criterion based on elevation visibility, SINR availability, and resource admissibility. The performance is evaluated in terms of handover success rate, signaling overhead, number of group handover actions, handover rate, and handover objective value J.

### 4.2. Simulation Results and Discussion

**Figure 3:**
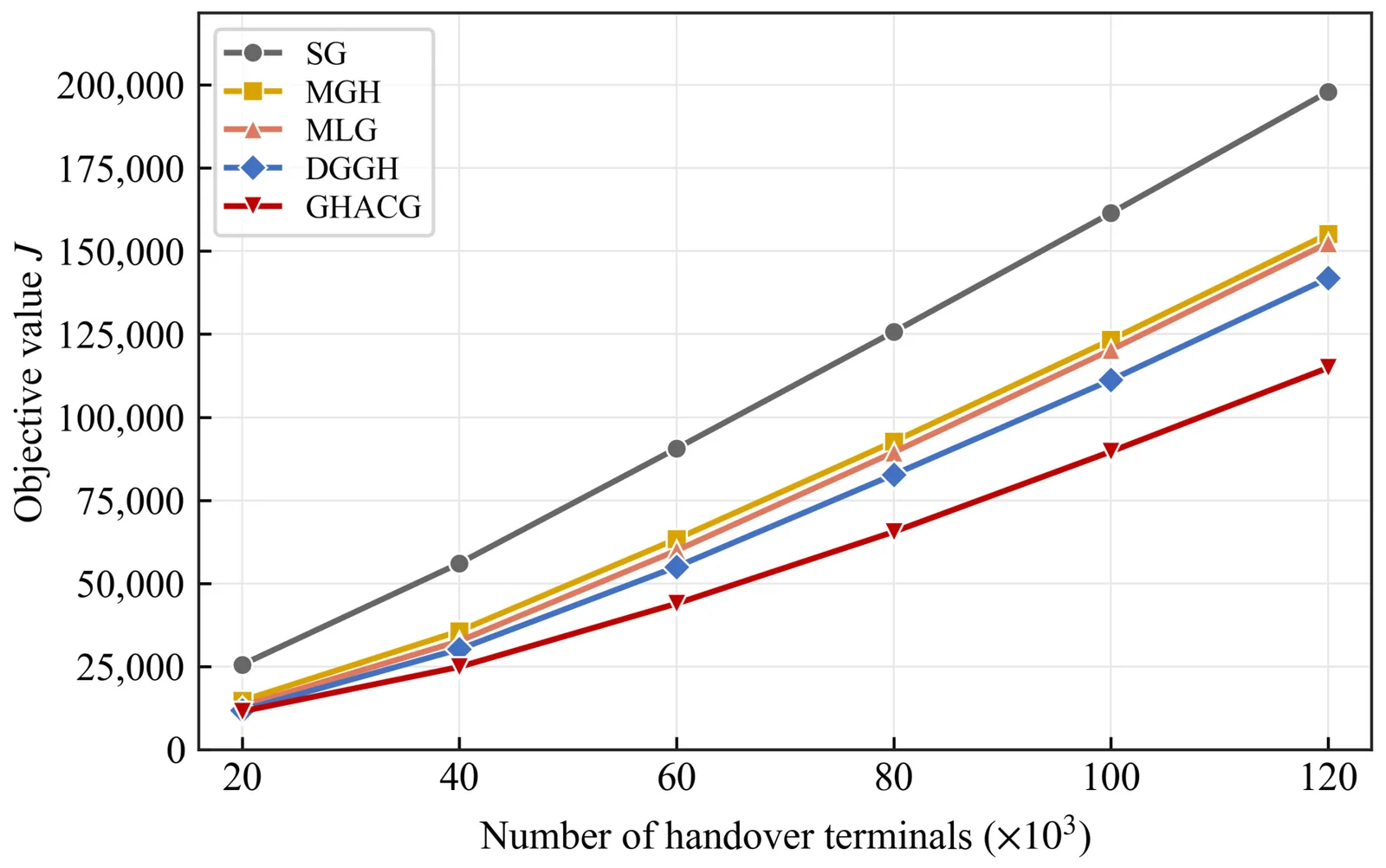
Objective value versus the number of terminals.

**Figure 4:**
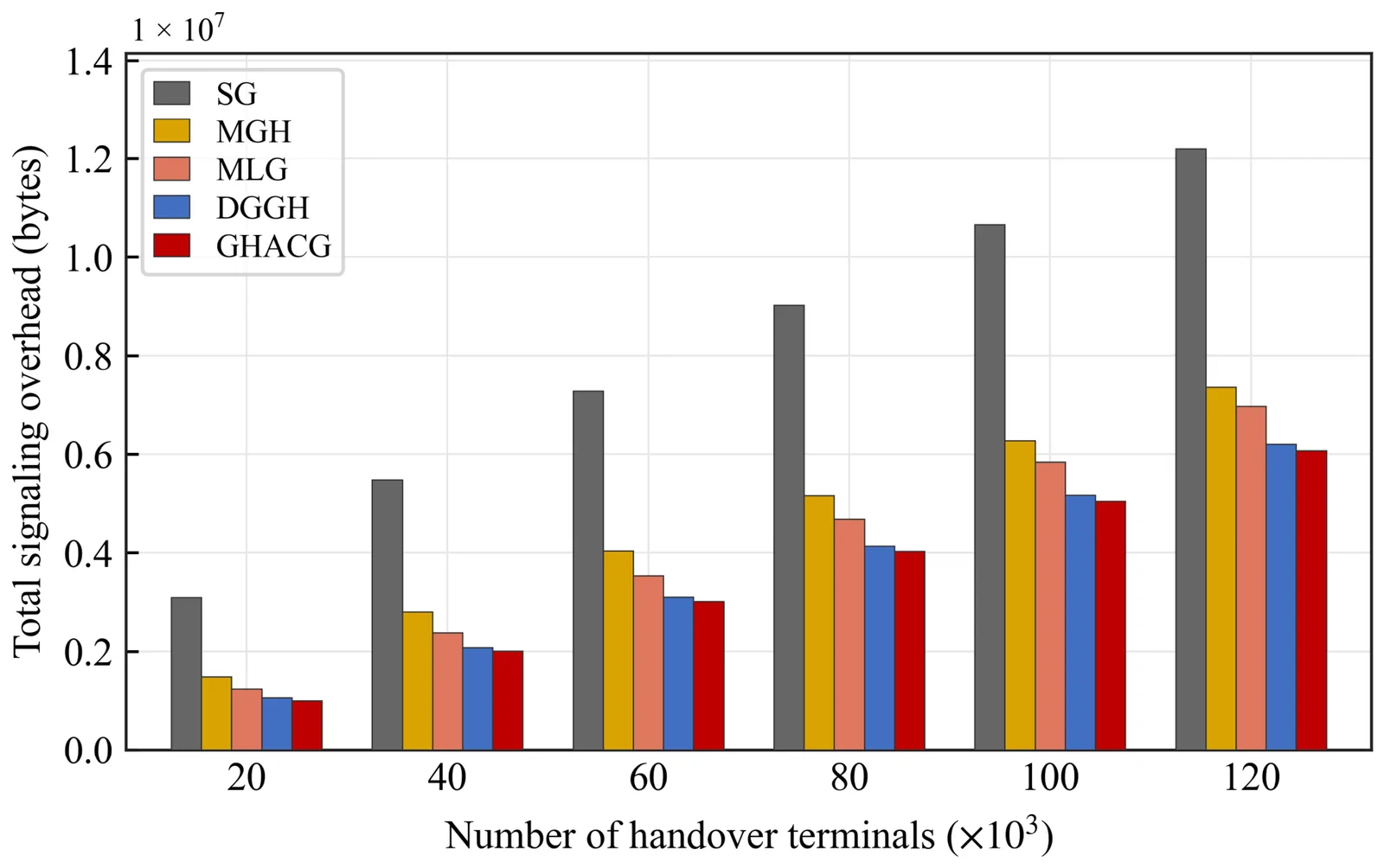
Total signaling overhead versus the number of terminals.

**Figure 5:**
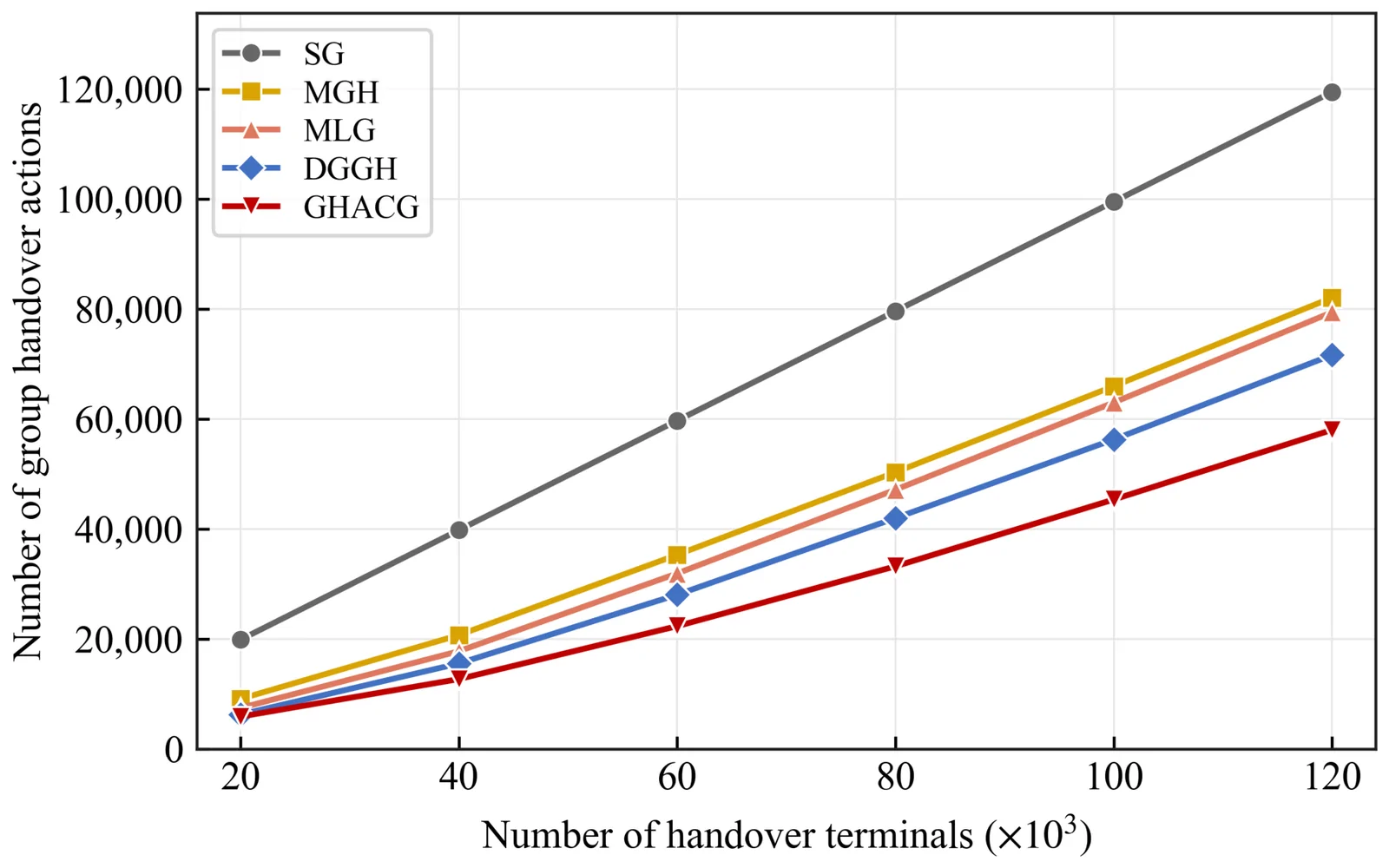
Number of handover actions versus the number of terminals.

**Figure 6:**
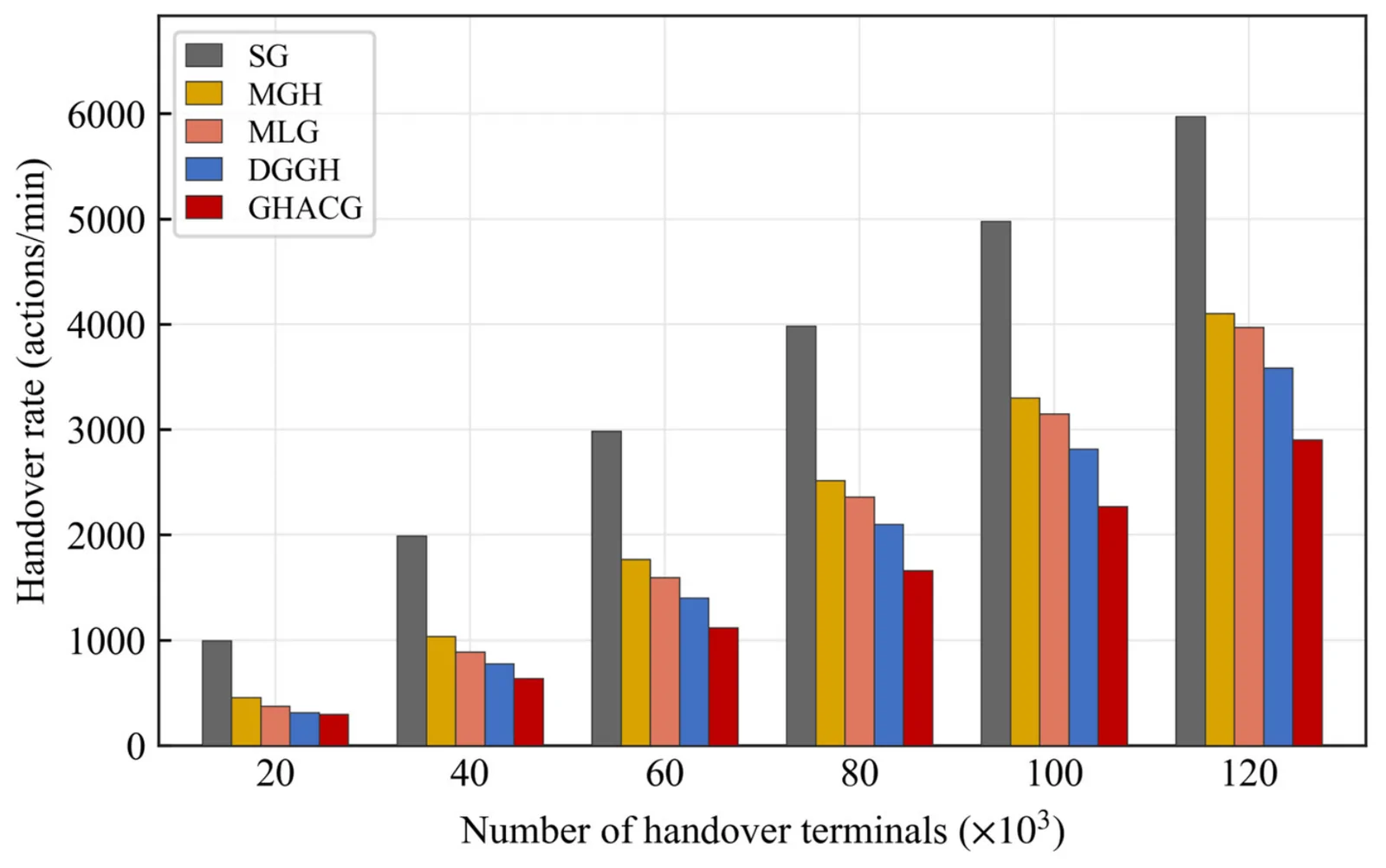
Handover rate versus the number of terminals.

**Figure 7:**
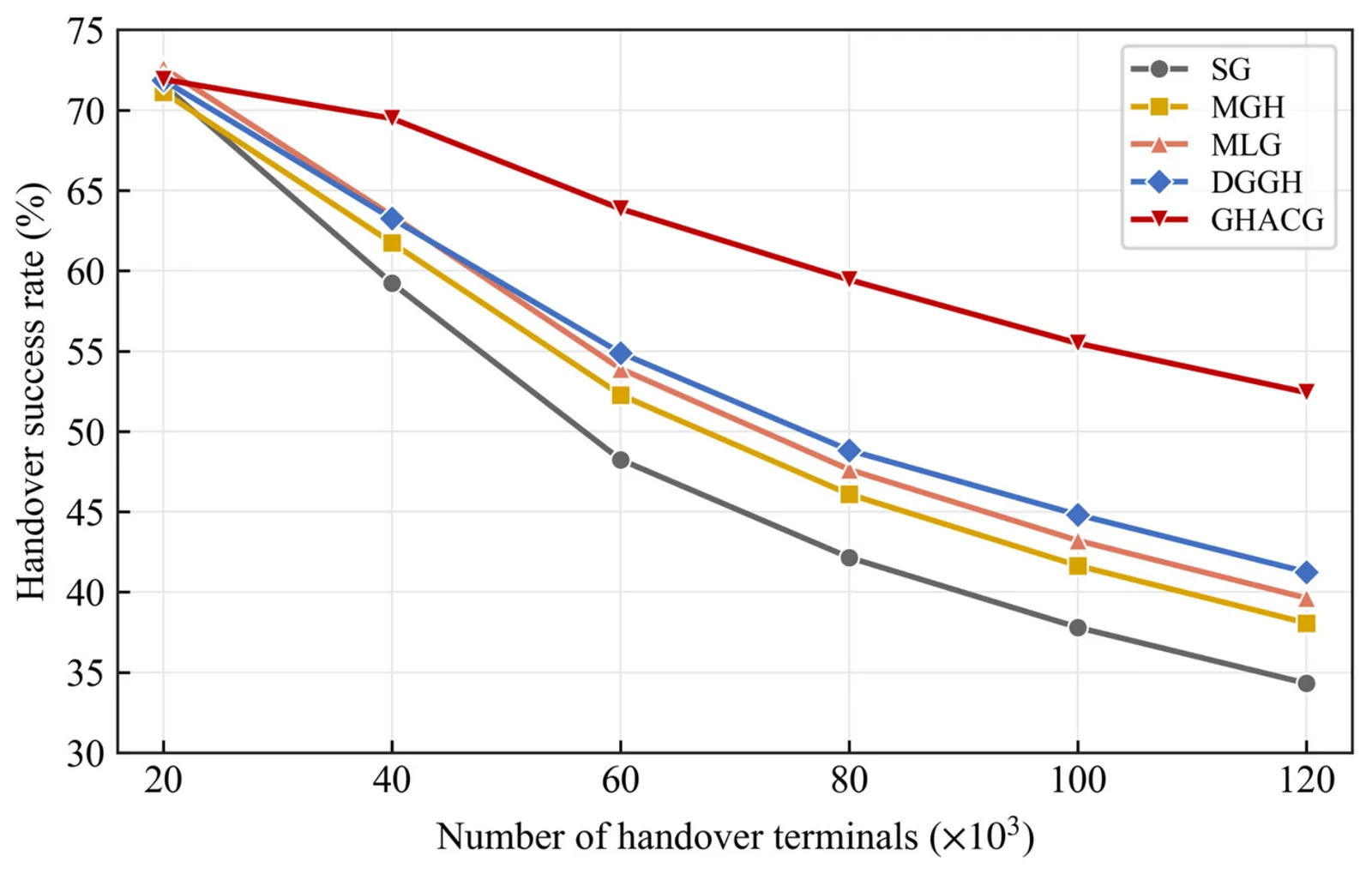
Handover success rate versus the number of terminals.

To eliminate the influence of the ground-user distribution on the statistical results, all experiments are conducted with five independent random seeds. Unless otherwise stated, the curves in Figure 3, Figure 4, Figure 5, Figure 6 and Figure 7 report the mean over five seeds. Because these fluctuations are relatively small, error bars are omitted from the figures. Among all compared schemes, the lowest and highest relative seed-to-seed fluctuations are as follows: from −0.04% to +1.94% for J, from −0.01% to +0.37% for the signaling overhead, from −0.01% to +1.90% for the number of group handover actions, from −0.01% to +1.90% for the handover rate, and from −0.09% to +0.85% for the handover success rate.

Figure 3 shows the handover objective value J under different numbers of handover terminals. Following the objective function in (20), J is defined as

(52) $$J=\sum_{a\in A}c_{a}x_{a}+P\sum_{i\in U^{ho}}z_{i}.$$

A smaller J indicates a lower overall handover cost. As the number of terminals increases, the objective values of all schemes increase due to heavier concurrent handover pressure. However, GHACG consistently achieves the lowest J among all compared schemes, demonstrating that the proposed method provides the best overall handover performance in large-scale concurrent handover scenarios.

Figure 4 compares the total signaling overhead of different schemes. As the number of handover terminals increases, the total signaling overhead increases for all schemes. The SG scheme produces the highest signaling overhead because each handover terminal independently triggers a complete handover procedure, and thus its control signaling overhead grows almost linearly with the number of terminals. In contrast, group handover schemes can aggregate part of the control signaling within each group handover action, thereby reducing repeated signaling transmission. Among the group handover schemes, GHACG achieves the lowest signaling overhead. This is because, compared with MGH, DGGH, and MLG, GHACG does not rely on a pre-grouping stage based on predefined user similarity information. Instead, it constructs executable group handover actions directly under target compatibility and resource admissibility constraints, which enables more efficient signaling aggregation and reduces the overall control overhead.

Figure 5 shows the number of handover actions generated by different schemes. For SG, each handover terminal corresponds to one handover action, and therefore SG produces the largest number of actions. Group handover schemes reduce the number of actions by allowing multiple terminals to be served in one group handover action. Among all group handover schemes, GHACG generates the fewest handover actions. This is because GHACG does not restrict terminal aggregation to groups obtained from predefined similarity metrics. Instead, it forms group handover actions according to whether terminals can be jointly served by the same feasible target satellite under the resource constraint. Therefore, a selected GHACG action can include more terminals that satisfy the same executable handover condition, thereby reducing the number of control actions required to handle the same handover demand.

Figure 6 reports the handover rate, measured by the number of handover actions per minute. This metric further reflects the time-domain control-plane pressure caused by handover operations. The results show that GHACG maintains the lowest handover rate among all schemes, indicating that it can effectively reduce the frequency of handover control operations and alleviate potential signaling congestion in large-scale concurrent handover scenarios.

Figure 7 shows the handover success rate under different numbers of terminals. As the number of terminals increases, the success rates of all schemes decrease due to the intensified competition for limited satellite access resources. Nevertheless, GHACG consistently outperforms all baseline schemes. When the number of terminals reaches $1.2\times 10^{5}$, GHACG improves the handover success rate by 11.17% compared with the best-performing baseline DGGH.

The signaling overhead, the number of group handover actions, the handover rate, and the handover success rate are jointly determined by the selected executable-action set. Under fixed pre-grouping, handover actions are restricted to predefined groups, so residual access bandwidth cannot be fully occupied by other feasible terminals. The idle resources leave more terminals unserved, which lowers the success rate and further increases the number of actions, the handover rate, and the repeated common signaling. GHACG is not restricted by a fixed grouping. By forming executable actions directly under target and resource compatibility, it utilizes the available bandwidth more fully and serves more terminals with fewer actions. The handover rate and the duplicated signaling therefore decrease, while the success rate increases. As shown in Figure 3, Figure 4, Figure 5, Figure 6 and Figure 7, GHACG achieves a higher success rate with lower signaling overhead, fewer actions, and a lower handover rate than the compared schemes, and thus attains better overall handover performance.

Table 3 reports the GHACG runtime, peak memory occupancy, number of generated columns, and number of column-generation iterations. From $2.0\times 10^{4}$ to $1.2\times 10^{5}$ terminals, the runtime increases from 1.05 s to 5.62 s, the peak memory occupancy remains between 9.0 MB and 43.6 MB, and the generated column pool increases from 517 to 1395. The column-generation process terminates after four iterations at every scale. These results show that the runtime and peak memory occupancy of GHACG remain acceptable for practical deployment at the tested terminal scales. The algorithm proposed in this paper is scalable and practical.

### 4.3. Pricing and Solution-Quality Validation

Two complementary validations were conducted. First, complete executable-action sets were enumerated for controlled small-scale instances to compare heuristic pricing with exact pricing at each iteration and examine whether the heuristic missed any negative-reduced-cost columns. Second, physically grounded instances with 300–600 terminals were used to evaluate pricing quality and final solution quality at larger scales.

Across the 15 small-scale instances, comprising five instances each with 8, 10, and 12 terminals, exact enumeration confirmed the existence of negative-reduced-cost columns in 74 pricing iterations. The heuristic detected such columns in 72 iterations, yielding a detection rate of 97.30%. The two missed iterations occurred in the 12-terminal instances, where the remaining negative columns had reduced costs of −0.25 and −0.50. One miss did not affect the final integer solution, whereas the other was associated with one additional failed terminal. Overall, the recovered integer solution matched the complete-action-space MILP optimum in 14 of the 15 instances.

For the second validation, we extracted smaller instances from the ephemeris-based scenario used in the main simulations to evaluate both the heuristic pricing process and the quality of the final integer solution. We selected two target satellite–slot resources with a common set of eligible terminals and sampled 300, 500, and 600 terminals from this set, with three instances at each scale. Their original links to the selected resources and corresponding bandwidth demands were retained. Since the nominal residual bandwidth caused no capacity-driven admission failures in these instances, we reduced the available bandwidth only for this validation to create resource competition. The same nine instances and capacity constraints were used in the pricing audit and the exact MILP comparison.

Within the second validation, we first evaluate the heuristic pricing process on these instances. At each audited GHACG iteration, the current restricted master problem is solved in its linear relaxation form, and the resulting dual prices are used by both pricing procedures. The heuristic pricing procedure generates candidate executable-action columns using the proposed greedy construction rule. For comparison, the pricing subproblem associated with each satellite–slot resource is formulated as a binary optimization problem and solved to optimality by a CPLEX-based exact pricing oracle. The exact oracle returns a minimum-reduced-cost feasible column for each resource.

An iteration is classified as an improving iteration if the CPLEX-based exact pricing oracle identifies at least one unseen executable-action column with a negative reduced cost. The heuristic pricing procedure is considered to detect an improving iteration if it generates at least one negative reduced-cost column under the same restricted master problem and dual solution. To further compare the quality of the generated columns, an exact-best match is recorded when the minimum reduced cost among the heuristic-generated columns equals the minimum reduced cost returned by the exact pricing oracle.

Across the nine larger-scale validation instances, 81 pricing iterations were audited, including 21, 30, and 30 iterations for the 300-, 500-, and 600-terminal instances, respectively. The exact pricing oracle identified an improving column in every audited iteration. The heuristic detected every improving iteration, and its minimum generated reduced cost equaled the exact pricing optimum in all 81 iterations. These results indicate that the heuristic generated high-quality improving columns in the tested instances.

All validation experiments were conducted on a computer equipped with an Intel Core i7-14650HX processor and 16 GB of RAM. IBM ILOG CPLEX 12.9.0 was used for the linear-programming solves within GHACG and for the exact MILP benchmark. The exact benchmark assigns terminals to eligible resources and determines the required number of size-limited actions under the same capacity and objective constraints. It is equivalent to optimizing over the complete executable-action set for these instances; it does not use the action columns generated by GHACG. CPLEX certified an optimal solution for all nine instances.

Table 4 compares the final GHACG solutions with the exact MILP solutions. The mean success rate of GHACG is at most 0.39% below the exact MILP, and the largest mean objective-value gap among the nine instances is 3.76%. The lower absolute success rates in this table result from the deliberately capacity-constrained validation setting: even the exact solutions cannot admit all terminals. These comparisons provide empirical evidence of solution quality, but do not constitute a general optimality guarantee for heuristic pricing.

### 4.4. Ablation Study

To quantify the contributions of the two main components of GHACG, an ablation study is conducted on the 60,000-terminal instance with seed 1. Three variants are compared: the complete GHACG, GHACG without dual-price-guided pricing, and GHACG without integer recovery. The second variant generates candidate actions without using the dual prices of the restricted master problem, whereas the third variant omits the integer action recovery procedure. All three variants use the same instance, capacity constraints, initialization, and termination settings. The action cost and failure penalty are set to $c_{a}=1$ and P=2, respectively. The reported metrics include the handover success rate, the number of failed terminals, the number of executed actions, and the objective value.

As shown in Table 5, removing dual-price-guided pricing or integer recovery lowers the handover success rate and increases the objective value. These results demonstrate that both dual-price-guided pricing and integer recovery contribute to the solution quality of GHACG in the tested large-scale scenario.

### 4.5. Sensitivity to Main Parameters

To evaluate the sensitivity of the proposed algorithm to the main parameters, GHACG is re-run on the same 60,000-terminal instance with seed 1. Six parameters are examined: the failure penalty P, residual bandwidth, SINR threshold, minimum elevation angle, traffic-demand scale, and decision-slot length. Table 6 reports the handover success rate and the number of scheduled actions for every case.

As shown in the table, when $P/c_{a}\geq 2$, the success rate and the number of actions stabilize. Residual bandwidth and traffic demand are resource-side constraints, and relaxing or tightening them affects the handover success rate to a limited extent. Changes in the SINR threshold, the minimum elevation angle, and the decision-slot length have a substantial effect on the handover success rate. The number of actions varies substantially with the SINR threshold and the minimum elevation angle, whereas the remaining parameters induce only limited changes in the action count.

### 4.6. Robustness Analysis Under Non-Ideal Channel Conditions

The nominal experiment uses LOS links. To examine NLOS conditions, each candidate link on the same 60,000-terminal instance is drawn as LOS or NLOS from the elevation-dependent rural/suburban Ka-band probability in 3GPP TR 38.811. Clutter loss and the NLOS shadow-fading standard deviation are applied only to the links drawn as NLOS. Table 7 reports the handover success rate and the number of scheduled actions.

Under NLOS conditions the handover success rate decreases, while the number of actions changes only slightly.

## 5. Conclusions

This paper investigated the large-scale group handover problem in LEO satellite networks and proposed a new paradigm for massive-user group handover. Different from conventional two-stage methods that first form terminal groups and then perform handover scheduling, this paper formulated group handover as an executable-action selection problem, where each action is composed of a terminal set, a target satellite, and a handover slot. Based on the proposed target compatibility and resource compatibility conditions, the executable group handover action space was constructed, and a signaling overhead model was further developed by distinguishing common control fields and terminal-specific fields in group handover signaling. To solve the resulting large-scale combinatorial optimization problem, a Group Handover Action Column Generation (GHACG) algorithm was proposed. The algorithm uses the dual prices of the restricted master problem to guide the generation of high-value executable action columns, and further applies integer action recovery to obtain a feasible group handover scheme. System-level simulations based on realistic satellite ephemeris data demonstrated that GHACG outperforms representative single-user and group handover baselines in terms of handover success rate, signaling overhead, number of group handover actions, handover rate, and handover objective value J.

This study still has several limitations. Terminals are treated as static within each decision window. The main experiments assume LOS links, a simplified traffic model, and a fixed residual access bandwidth. Extending the evaluation to mobile terminals, time-varying residual bandwidth, and constellation-parameter variation is left for future work.

## Figures and Tables

**Table 1:** Summary of representative handover approaches in LEO satellite networks.

Technical Approach	Decision Entity	Main Methods
Rule-based and geometry-based	Individual user	Geometric criteria, threshold-based decisions, and stochastic geometry analysis [10,11,12,13]
Multi-attribute decision-making	Individual user	Preference-matrix filtering, weighted scoring, and TOPSIS ranking [14,15,16]
Graph-based optimization	Individual user/User group	Bipartite matching, minimum-cost maximum flow on multi-layer graphs, and integer programming [17,18,19,46,48]
Integer programming	User group	Sequential integer linear programming [47]
Dynamic programming	Individual user	MDP modeling and Bellman value iteration [20]
Metaheuristic optimization	Individual user/User group	Simulated annealing [49,50]
Mathematical programming	Users and access resources	Lyapunov optimization, alternating optimization, and Lagrangian dual methods [33,38,45]
Clustering-based grouping handover	User group	Hierarchical clustering, beam-limited hierarchical clustering, and adaptive spectral clustering [41,42,43]
Game-theoretic optimization	User group	Correlation-based prediction, congestion games, and best-response iteration [44]
Reinforcement learning	Individual user/User group/Relay node	DQN/DDQN, Actor-Critic, PPO, and multi-agent TD3 [21,22,23,24,25,26,27,28,29,30,31,32,36,37,39,51,52]
Hybrid optimization	Users and access resources	Graph matching with particle swarm optimization; Riemannian manifold optimization with multi-agent deep Q-learning [34,35]

**Table 2:** Main simulation parameters.

Parameter	Value
Number of satellites	1584
Number of orbital planes	72
Number of satellites per plane	22
Orbital altitude	550 km
Inclination	53°
Satellite speed	7.5 km/s
Ephemeris sampling interval	1 s
Decision slot length Δt	30 s
Carrier frequency $f_{c}$	20 GHz
Channel bandwidth B	400 MHz
Satellite EIRP ${EIRP}_{s}$	30 dBW
Terminal antenna gain $G_{i}$	39.7 dBi
Terminal noise figure $F_{i}$	1.2 dB
Noise power spectral density $N_{0}$	−174 dBm/Hz
Minimum elevation angle $e_{min}$	30°
Minimum remaining service time $T_{min}^{rem}$	180 s
SINR threshold $\gamma_{min}$	10 dB
Shadow fading standard deviation $\sigma_{SF}$	0.4–3.6 dB
Clutter loss $L_{i,s}^{CL}\left(\tau \right)$	0 dB
Zenith atmospheric attenuation $A_{zenith}\left(20\;GHz\right)$	0.26 dB
Tropospheric scintillation loss $L_{i,s}^{scin}\left(\tau \right)$	0.12–0.30 dB
Residual access bandwidth $C_{s}\left(\tau \right)$	1500 MHz
Service rate requirement $R_{i}^{req}$	$\left\{2,5,10\right\}$ Mbps
Probability of $R_{i}^{req}$	$\left\{0.4,0.4,0.2\right\}$
Measurement report size $C_{meas}$	16 Bytes
Handover request size $C_{req}$	37 Bytes
Handover request ACK size $C_{ack}$	37 Bytes
RRC reconfiguration size $C_{recfg}$	37 Bytes
Handover complete size $C_{complete}$	37 Bytes
Number of random seeds	5
Action cost $c_{a}$	1
Handover failure penalty P	2
Reduced-cost tolerance ϵ	$10^{-7}$

Note: The signaling message sizes follow the representative values used in [46].

**Table 3:** Computational performance of GHACG at different terminal scales.

Terminals	Runtime (s)	Peak Memory (MB)	Generated Columns	Iterations
20,000	1.05	9.0	517	4
40,000	1.71	17.3	770	4
60,000	2.55	24.7	959	4
80,000	3.31	29.0	1119	4
100,000	4.14	41.7	1264	4
120,000	5.62	43.6	1395	4

**Table 4:** Comparison of GHACG with the exact MILP solutions.

Terminals	Exact MILP Success Rate (%)	GHACG Success Rate (%)	Objective Gap (%)	RMP LP-to-Integer Gap (%)	GHACG Runtime (s)	Exact MILP Time (s)
300	89.44 ± 0.19	89.11 ± 0.19	3.16 ± 0.06	2.87 ± 0.63	0.28 ± 0.04	0.33 ± 0.16
500	89.60 ± 0.00	89.27 ± 0.12	3.21 ± 1.11	1.40 ± 1.12	0.40 ± 0.09	0.42 ± 0.07
600	89.67 ± 0.00	89.28 ± 0.10	3.76 ± 0.93	1.66 ± 0.32	0.47 ± 0.16	3.37 ± 0.32

**Table 5:** Ablation results of GHACG on the 60,000-terminal instance.

Variant	Success Rate (%)	Actions	Objective J
Complete GHACG	63.84	767	43,957
Without dual-price-guided pricing	57.50	665	51,433
Without integer recovery	57.73	667	51,155

**Table 6:** Sensitivity of GHACG on the 60,000-terminal instance.

Parameter	Values	Success Rate (%)	Number of Actions
Failure penalty P	0.1/1/**2**	63.51/63.83/**63.84**	716/758/**767**
Residual bandwidth (MHz)	1125/**1500**/1875	59.23/**63.84**/67.26	716/**767**/803
SINR threshold (dB)	8/**10**/12	89.36/**63.84**/38.43	1036/**767**/487
Minimum elevation (°)	20/**30**/40	95.89/**63.84**/13.99	988/**767**/188
Traffic-demand scale	0.75/**1**/1.25	68.23/**63.84**/60.34	811/**767**/729
Decision-slot length (s)	15/**30**/60	80.62/**63.84**/58.17	960/**767**/659

**Table 7:** Robustness of GHACG under mixed LOS/NLOS conditions.

Scenario	Success Rate (%)	Number of Actions
Nominal (LOS)	63.84	767
Mixed LOS/NLOS	61.91	749

## Data Availability

All results supporting the conclusions of this work are included within the article. No additional datasets were generated for public deposition.
